# A bovine antibody possessing an ultralong complementarity-determining region CDRH3 targets a highly conserved epitope in *sarbecovirus* spike proteins

**DOI:** 10.1016/j.jbc.2022.102624

**Published:** 2022-10-20

**Authors:** Matthew J. Burke, James N.F. Scott, Thomas C. Minshull, Zeqian Gao, Iain Manfield, Sinisa Savic, Peter G. Stockley, Antonio N. Calabrese, Joan Boyes

**Affiliations:** 1 School of Molecular and Cellular Biology, Faculty of Biological Sciences, University of Leeds, Leeds, United Kingdom; 2 Astbury Centre for Structural Molecular Biology, Faculty of Biological Sciences, University of Leeds, Leeds, United Kingdom; 3 National Institute for Health Research, Leeds Biomedical Research Centre and Leeds Institute of Rheumatic and Musculoskeletal Medicine, Wellcome Trust Brenner Building, St James's University Hospital, Leeds, United Kingdom

**Keywords:** antibody, antibody engineering, antiviral agent, epitope mapping, hydrogen-deuterium exchange, bNAb, broadly neutralizing antibody, FACS, fluorescence-activated cell sorting, gDNA, genomic DNA, HDX-MS, hydrogen deuterium exchange mass spectrometry, IMAC, immobilized metal affinity chromatography, LV, lentivirus, NTD, N-terminal domain, RBD, receptor-binding domain, SPR, surface plasmon resonance, TU, transduction Units, VOC, variant of concern

## Abstract

Broadly neutralizing antibodies have huge potential as novel antiviral therapeutics due to their ability to recognize highly conserved epitopes that are seldom mutated in viral variants. A subset of bovine antibodies possess an ultralong complementarity-determining region (CDR)H3 that is highly adept at recognizing such conserved epitopes, but their reactivity against *Sarbecovirus* Spike proteins has not been explored previously. Here, we use a SARS-naïve library to isolate a broadly reactive bovine CDRH3 that binds the receptor-binding domain of SARS-CoV, SARS-CoV-2, and all SARS-CoV-2 variants. We show further that it neutralizes viruses pseudo-typed with SARS-CoV Spike, but this is not by competition with angiotensin-converting enzyme 2 (ACE2) binding. Instead, using differential hydrogen-deuterium exchange mass spectrometry, we demonstrate that it recognizes the major site of vulnerability of *Sarbecoviruses*. This glycan-shielded cryptic epitope becomes available only transiently *via* interdomain movements of the Spike protein such that antibody binding triggers destruction of the prefusion complex. This proof of principle study demonstrates the power of *in vitro* expressed bovine antibodies with ultralong CDRH3s for the isolation of novel, broadly reactive tools to combat emerging pathogens and to identify key epitopes for vaccine development.

Our societies live with the constant threat that a deadly, highly transmissible new pathogen emerges and spreads rapidly due to extensive global travel, high population densities, and negligible pre-existing immunity. Although none of the three recently transferred coronaviruses was both highly transmissible and highly fatal, should such a virus emerge, the socioeconomic consequences would be devastating. The fact that huge reservoirs of coronaviruses are present in species, such as bats, implies that the threat of new coronavirus spill-over events is significant. Perhaps more worryingly, zoonotic transfer of other pathogens has also been occurring at an accelerated rate in recent years. Likewise, viruses such as West Nile Fever, for which no specific treatments exist, are spreading more widely due to global warming. Whilst vaccines proved remarkably successful at slowing SARS-CoV-2 transmission and preventing severe disease (1, 2, 3), they are not a panacea due to their typically high failure rate, potentially low efficacy, and risk that they may not protect against all new variants. Moreover, not all individuals mount an effective immune response to vaccines. It is therefore vital to develop complementary therapeutics that can be rapidly deployed in response to new pathogenic threats.

Monoclonal antibodies (mAbs) have proved to be effective alternative treatments, particularly for individuals with weakened immune systems (4) but they too suffer from the fact that new virus variants can emerge to escape the protection that they previously conferred. Indeed, in the case of SARS-CoV-2, the Omicron variant, with 15 mutations in the receptor-binding domain (RBD), was resistant to neutralization by almost all of the original mAb therapeutics (5, 6).

By contrast, broadly neutralizing antibodies (bNAbs) target conserved epitopes that are often of functional importance to the virus and therefore are much less vulnerable to escape mutations. Given that such epitopes are also likely to be found on related, emerging pathogens, considerable effort is being expended to identify such antibodies (7, 8, 9). Whilst humans can generate bNAbs, this normally requires repeated pathogen exposure, either *via* chronic infection or recurrent infections and/or vaccinations (10, 11, 12). Remarkably, however, cattle are highly adept at generating bNAbs. Around 10% of bovine antibodies possess an ultralong CDRH3 of 40 to 71 aa that extends up to 40 Å away from the main immunoglobulin fold to reach otherwise occluded epitopes, including those obscured by large glycan moieties. This CDRH3 forms an extended β-strand stalk supporting a disulfide-bonded ‘knob’ domain (13, 14, 15) where the latter is entirely responsible for all direct antigen interactions. By sitting on top of a long β-stranded stalk, it can reach epitopes that are normally occluded within deep clefts or crevices (16, 17) and that are unreachable by typical antibodies.

The bovine system has been used to great effect to isolate bNAbs against HIV-1 from a cow immunized with a stabilized HIV-1 Env (16). The highest affinity antibody (NC-Cow-1) engages with the CD4 binding site of Env and potently neutralizes a large panel of HIV-1 variants (16). The remarkable breadth of this nAb can be attributed to its unconventional paratope structure that allows it to target a small footprint on Env and reduce its vulnerability to escape mutations (17). Similarly, broad and potent nAbs with an ultralong CDRH3 have been isolated against Foot-and-mouth disease virus from infected cattle (18). Crucially, the paratope from NC-Cow-1 was successfully transferred to a human antibody scaffold with minimal loss of activity, establishing the feasibility of using humanized antibodies with an ultralong bovine CDRH3 as therapeutic tools (19).

Immunization of cattle, however, results in high titers of potent bNAbs only after multiple booster immunizations and a period of several months (16), a timeframe that may be too lengthy to prevent significant mortality from a deadly new pathogen. By contrast, the entire repertoire of bovine ultralong CDRH3-containing antibodies is encoded by recombination of the same three gene segments: V_H1-7_, D_H8-2_, and J_H2-4_, where D_H8-2_ is primarily responsible for encoding the disulfide-bonded knob domain (15, 20, 21). This exclusive use of gene segments lends itself well to the specific isolation of ultralong CDRH3 sequences (Fig. 1*A*) and the generation of libraries that can be screened for binding to antigens from emerging pathogens.

**Figure 1:**
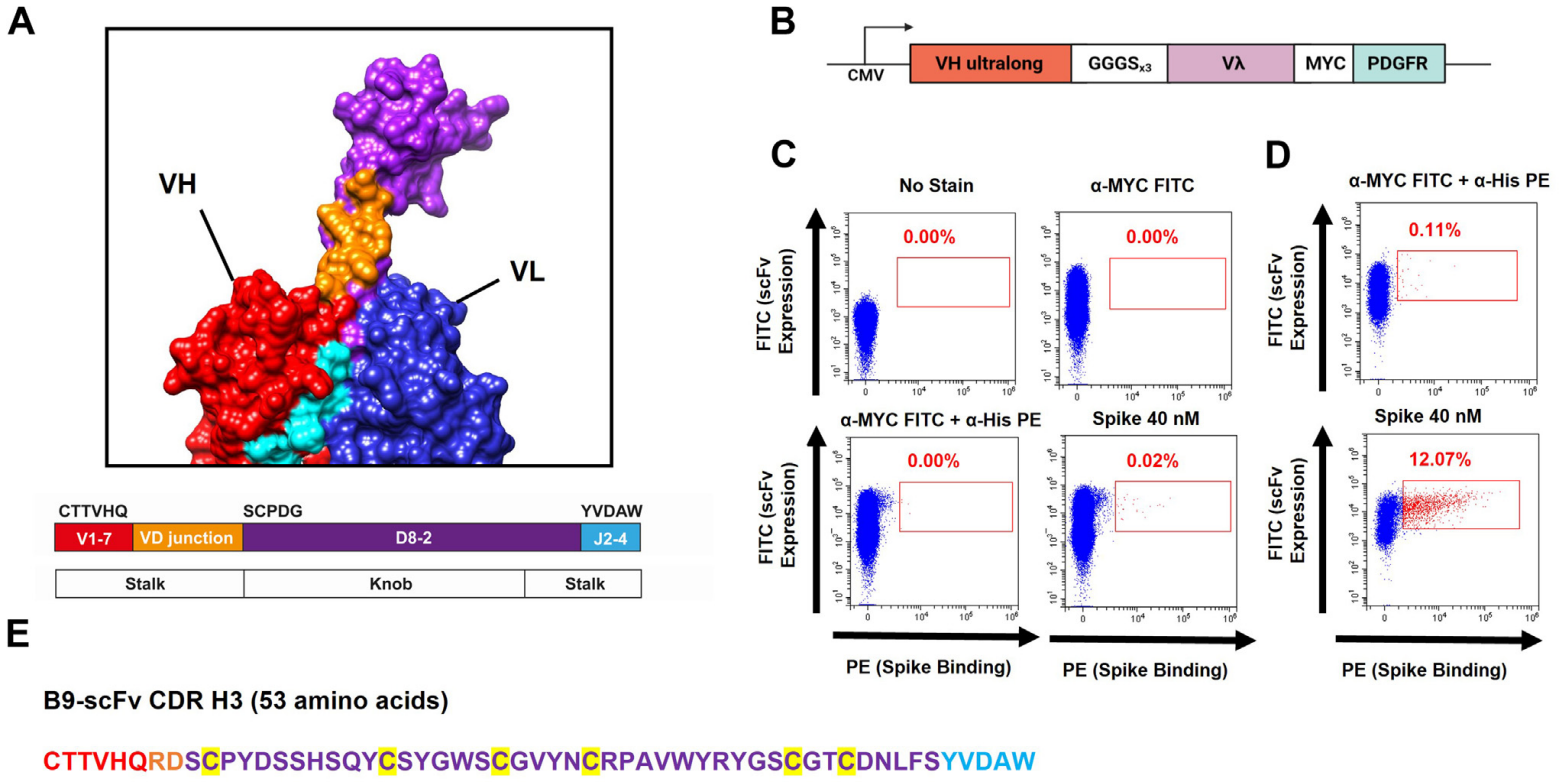
**Cell display and binding of ultralong scFvs to SARS-CoV-2 Spike protein.***A*, structure of an ultralong CDRH3 stalk and knob domain (PDB: 4K3D) color coded to show the parts encoded by the V_H1-7_, D_H8-2_, and J_H2-4_ gene segments. *B*, construct used to express membrane-bound scFvs with an ultralong V_H_ under the control of the cytomegalovirus promoter (CMV). The C-terminal Myc tag and platelet-derived growth factor receptor transmembrane domain (PDGFR; for cell surface expression) are shown. *C*, 293T cells were transfected with round 0 scFv library. *Upper*: FACS plots of these cells without (*left*) and with (*right*) incubation with α-Myc-FITC. *Lower*: FACS plots showing α-His-PE and α-Myc-FITC staining in the absence (*left*) or presence (*right*) of 40 nM Spike. The *red box* indicates cells expressing scFvs that bind Spike. *D*, enrichment of Spike-binding scFvs after two rounds of plasmid-based selection. 293T cells were transfected with round 2 plasmid library incubated without (*upper*) and with (*lower*) 40 nM Spike. The *red box* shows cells expressing scFvs that bind Spike. *E*, the amino acid sequence of the Spike-binding B9-scFv. The regions encoded by V_1-7_ (*blue*), the VD junction (*orange*), D_8-2_ (*dark gray*), and J_2-4_ (*green*) are shown. Cysteine residues are highlighted in *yellow*. FACS, fluorescence-activated cell sorting.

Here, we sought to isolate ultralong bovine heavy chains that bind to SARS-CoV-2 and related coronaviruses in a proof of principle study. We capitalized on previous findings that ultralong CDRH3s pair with a relatively invariable Vλ light chain (15, 20), to generate a single chain variable fragment (scFv) scaffold into which ultralong heavy chain–only libraries can be cloned and expressed. Using mammalian cell surface display and His-tagged SARS-CoV-2 Spike glycoprotein, we isolated an ultralong scFv (B9-scFv) from a SARS-CoV-2-naïve heavy chain library that binds to SARS-CoV-2 RBD, all current SARS-CoV-2 variants, and notably, also to SARS-CoV RBD. B9-scFv does not compete with Spike binding to its cell surface receptor, ACE2, but instead neutralizes SARS-CoV pseudotyped lentiviruses (LVs), likely by destabilizing the prefusion complex. Consistent with this, the epitope localizes to a cryptic cleft on the inner face of the RBD that is thought to be only transiently accessible. This site overlaps with the footprints of some of the broadest anti-SARS-CoV-2 antibodies identified to date (7D6/6D6 (22), FD20 (23), and S2H97 (7)) and corresponds to the main site of vulnerability of *Sarbecoviruses* (24). Remarkably, this broadly reactive bovine CDRH3 was isolated from a library of only modest sequence diversity. This attests to the huge potential of the bovine system as a source of broadly active antibodies that can protect against emerging pathogens and their variants.

## Results

### Cell surface display of bovine ultralong scFvs

To establish a cell surface display platform to screen ultralong CDRH3 libraries, we first amplified the variable exons from the leukocyte genomic DNA (gDNA) of two adult cows to generate a library of ultralong bovine paratopes. The ultralong CDRH3s were then enriched by nested PCR and size selection, to produce an initial heavy chain library with >96% ultralong CDRH3s (Fig. S1*A*). These purified amplicons were inserted into the pBovShow expression cassette (Fig. 1*B*), resulting in them being joined to an invariant Vλ light chain variable domain (Vλ-LC) *via* a flexible linker. Following transient transfection into 293T cells and screening by flow cytometry, over 70% of the ultralong scFv clones were found to be expressed on the cell surface (Figs. 1*C* and S1*B*). This suggests that the invariant Vλ variable domain pairs with most ultralong heavy chains, even in an scFv format and further implies that this is an effective scaffold for the expression of libraries of ultralong bovine CDRH3 for affinity-based panning.

### Isolation of a bovine ultralong scFv that binds to the SARS-CoV-2 Spike glycoprotein

Next, we screened our ultralong scFv library for binding to the recombinant full-length SARS-CoV-2 Spike glycoprotein, with two stabilizing proline mutations (S-2P; (25)) by transient transfection of the library into 293T cells and subsequent flow cytometry. Initially, a small population of cells expressing scFvs that bind Spike protein was isolated (0.02%; Fig. 1*C*); these were enriched by two further rounds of plasmid recovery, retransfection, and flow cytometry (Fig. 1*D*), resulting in a sharp increase in the proportion of Spike-binding scFvs (Fig. 1*D*). Sequencing the scFv library at different stages of enrichment allowed the original library diversity to be estimated at <1 × 10^4^ unique sequences (Fig. S1*C*). While the number of unique ultralong CDRH3 sequences recovered here is low, it is comparable to the numbers isolated previously from two cows (15). Despite the small size of the present library, the marked enrichment of Spike-binding scFvs encouraged further investigation.

Therefore, to more efficiently isolate the Spike-binding scFv(s), we cloned our enriched library into LV vectors (Fig. S2*A*) and generated LV particles pseudotyped with VSV-G. These were transduced into 293T cells at a low titer to achieve few integrated scFv sequences per cell. After puromycin selection and expansion, only 0.42% of the transduced cells bound Spike protein (Fig. S2*B*), and from this population, we isolated 15 single cell clones that interacted with 40 nM Spike protein. Three of these single cell clones harbored a single scFv, and remarkably, the nucleotide sequence of all three scFvs was identical. This sequence, termed B9-scFv (Fig. 1*E*), encodes a 53 aa ultralong CDRH3 that interacts with the SARS-CoV-2 Spike (Fig. S2*B*, lower). B9-scFv accounted for 53% of all scFvs from the LV-transduced cells after a single selection for Spike binding, and this increased to 83% upon a further round of enrichment by flow cytometry, suggesting that the ultralong B9-scFv accounts for much the anti-Spike activity in our library.

### The epitope for B9-scFv is within the SARS-CoV-2 RBD

**Figure 2:**
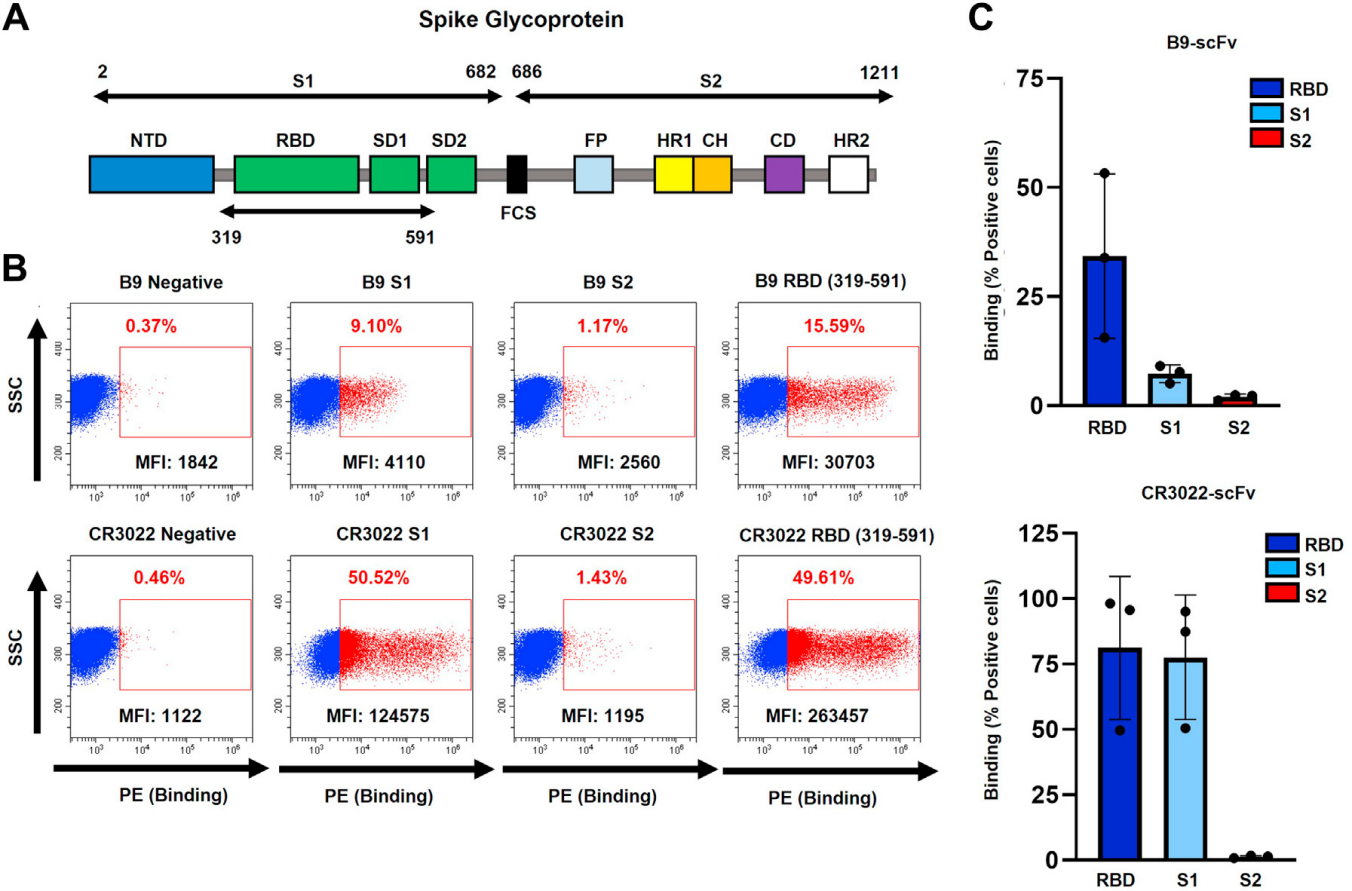
**Isolation of an ultralong scFv that binds to WT SARS-CoV-2 Spike receptor-binding domain.***A*, cartoon depicting the SARS-CoV-2 Spike protein subdomains across aa 2 to 1211. NTD: N-terminal domain, RBD: receptor-binding domain, SD1: sub-domain 1, SD2: sub-domain 2, FCS: furin cleavage site, FP: fusion peptide, HR1: heptad repeat 1, CH: central helix, CD: connector domain, HR2: heptad repeat 2. *B*, FACS plots of 293T cells transfected with plasmids encoding B9-scFv (*top panels*) or CR3022-scFv (*bottom panels*) and incubated with or without 2 μM of the 8xHis-tagged SARS-CoV-2 subdomains shown. SSC refers to side scatter. *C,* relative binding of cell surface–expressed scFvs to Spike subdomains summated from FACS experiments shown in (*B*). Relative binding was calculated as the fold enrichment in percent positive cells from scFv transfected cells incubated with the indicated subdomain, compared to percent positive, nontransfected cells incubated with the same subdomain. Data are presented as mean ± SD (n = 3). Lower binding to the S1 domain compared to the RBD may be due to occlusion of the epitope in S1. FACS, fluorescence-activated cell sorting.

To begin to locate B9-scFv’s epitope, we purified the SARS-CoV-2 Spike subdomains (Fig. 2*A*), S1 (aa 2–682), S2 (aa 686–1211), and RBD-SD1 (aa 319–591) *via* immobilized metal affinity chromatography (IMAC; Fig. S3*A*). Cells transiently expressing B9-scFv display clear binding to Spike (Fig. S3*B*), the RBD, and to a lesser extent, the S1 domain (Figs. 2, *B*, *C* and S3*B*). Importantly, B9-scFv did not interact with equivalent concentrations of the S2 domain (Fig. 2, *B* and *C*). These data therefore localize the binding site of B9-scFv to RBD residues 319 to 591.

### B9-scFv is resistant to receptor-binding motif mutations

Bovine ultralong CDRH3s typically recognize conserved epitopes. If this is the case for B9-scFv, we would expect its binding will be unaffected by mutations in any of the SARS-CoV-2 variants of concern (VOC). To investigate this, we established an assay to monitor B9-scFv binding to cell surface–expressed Spike protein variants, and to this end, His-tagged B9-scFv was firstly expressed and purified from 293T cells (Fig. S4*A*). An expression vector for SARS-CoV-2 Spike (Wu-Hu-1 + D614G) was then transiently transfected into 293T cells and cell surface Spike expression was confirmed by staining with a positive control scFv (CR3022-scFv), which binds to an RBD epitope that is conserved between SARS-CoV-2 and SARS-CoV (26) (Fig. S4*B*, upper). Further control experiments verified concentration-dependent binding of purified B9-scFv to Spike-transfected cells and demonstrated that this is enriched compared to a negative control bovine ultralong scFv (137-scFv; Fig. S4*B*). Notably, purified B9-scFv demonstrated no reactivity with nontransfected cells, even when incubated at high concentrations (5 μM) for prolonged periods (1 h), strongly suggesting that its interaction with the Spike protein is specific.

**Figure 3:**
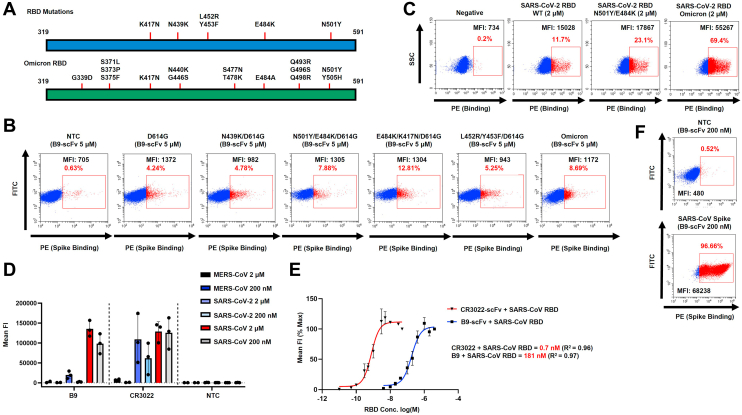
**Purified B9-scFv is broadly reactive and binds strongly to SARS-CoV RBD.***A*, cartoon depicting the positions of the mutations in the SARS-CoV-2 VOC Spike RBD (aa 319–591; *upper*) and Omicron VOC RBD (*lower*). All mutations are on the Wuhan-Hu-1 + D614G background. *B*, FACS plots showing B9-scFv (5 μM) binding to the indicated mutant SARS-CoV-2 Spike proteins expressed on the 293T cell surface. B9-scFv binding is indicated by the *red box*. *C*, FACS plots showing RBD variants (2 μM) binding to nontransfected 293T cells (NTC) and 293T cells transfected with B9-scFv. RBD binding is indicated by the *red box*. SSC refers to side scatter. *D*, FACS-based quantification of binding of B9-scFv or CR3022-scFv to MERS-CoV, SARS-CoV-2, or SARS-CoV RBD (Urbani variant). Mean fluorescence intensity (MFI) of PE staining was used to quantify binding (n = 3). Data are presented as mean ± SD. *E*, FACS-based half-maximal binding estimates of B9-scFv or CR3022-scFv binding to SARS-CoV RBD (Urbani variant). Data points are plotted as a percentage of the MFI obtained at the highest RBD concentration tested in each case (n = 2). Data are presented as mean ± SD. Estimated *K*_*D*_ and R^2^ values are indicated. *F*, FACS plots showing binding of purified B9-scFv (200 nM) to 293T cells that were nontransfected (*upper*) or transfected with full-length SARS-CoV Spike (Urbani variant; *lower*). FACS, fluorescence-activated cell sorting, RBD, receptor-binding domain; VOC, variants of concern.

Using this assay, we next examined B9-scFv binding to SARS-CoV-2 Spike protein variants. Crucially, binding is maintained to all of the mutations in the commonly circulating SARS-CoV-2 variants, including D614G, N501Y, E484K, Y453F, L452R, and K417N from the Alpha, Beta, Gamma, Delta, and Omicron variants (Fig. 3, *A* and *B*). Not only does this imply B9-scFv recognizes SARS-CoV-2 Spike glycoprotein in its native state but also suggests B9-scFv is broadly reactive.

To further confirm B9-scFv binding to the SARS-CoV-2 VOC, we purified SARS-CoV-2 RBDs (aa 319–591) harboring various mutations and incubated these with 293T cells expressing B9-scFv on the cell surface. Consistent with the data in Figure 3*B*, B9-scFv binds to RBDs carrying the mutations associated with the Alpha lineage as well as to the RBD of the hypermutated Omicron variant, that encompasses mutations found in the Beta and Gamma variants, with no significant loss of affinity compared to WT Spike (Fig. 3*C*). These data therefore strengthen the idea that B9-scFv binds to a conserved epitope.

### B9-scFv binds to SARS-CoV with nanomolar affinity

Given its potential broad reactivity, we next sought to determine if B9-scFv binds to other beta-coronavirus RBDs. The corresponding sequences of SARS-CoV RBD (aa 319–591) and MERS-CoV RBD (aa 368–586) were therefore cloned upstream of 8xHis tag and the proteins purified from 293T cells by IMAC (Fig. S4*C*). Surprisingly, the interaction between cell surface expressed B9-scFv and SARS-CoV RBD was markedly stronger than that observed with equivalent amounts of SARS-CoV-2 RBD. Maximal detectable binding by fluorescence-activated cell sorting (FACS) was observed at 2 μM SARS-CoV RBD and this was only partially reduced at 200 nM. By contrast, the interaction with SARS CoV-2 RBD was modest at 2 μM RBD (Fig. 3*D*). Both B9-scFv and the SARS-specific human CR3022-scFv were relatively unreactive with the MERS-CoV RBD at all concentrations tested (Fig. 3*D*), suggesting B9-scFv has specificity for SARS-CoVs.

The small difference in binding of B9-scFv to 2 μM and 200 nM SARS-CoV RBD indicates a nanomolar affinity. To further investigate this, binding of cell surface–expressed B9-scFv to a range of RBD concentrations (10 nM – 4 μM) was measured (Fig. 3*E*), which allowed an approximate *K*_*D*_ of 181 nM to be calculated. Using this same method, we approximated the *K*_*D*_ for CR3022-scFv interactions with SARS-CoV (0.7 nM) and SARS-CoV-2 (33 nM) RBD (Figs. 3*E* and S4*D*) values that are comparable to those reported in the literature (27). In order to estimate the *K*_*D*_ for B9-scFv binding to SARS-CoV-RBD using an orthogonal technique, we carried out surface plasmon resonance (SPR). A 1:1 fit of the SPR data yielded a *K*_*D*_ of 169 ± 22 nM, which is very close to that determined by flow cytometry. The binding of B9-scFv to SARS-CoV-2 RBD, however, is weaker and the affinity of the interaction could not be estimated by either method (Fig. S4*D*). This weaker binding is, however, consistent with previous studies that showed increased breadth of binding correlates with lower affinity (28, 29).

We next tested if purified B9-scFv recognizes the SARS-CoV RBD in the context of the Spike trimer. Consistent with our previous results, B9-scFv binds more strongly to cells expressing the SARS-CoV Spike than SARS-CoV-2 Spike, as lower concentrations of B9-scFv (<200 nM) are needed to label cells expressing this glycoprotein (Fig. 3*F*). Collectively, these data suggest that the bovine ultralong CDRH3 in B9-scFv crossreacts with viruses in the *Sarbecovirus* subgenus and binds SARS-CoV with a higher affinity than SARS-CoV-2.

### B9-scFv neutralizes SARS-CoV pseudotyped viruses

**Figure 4:**
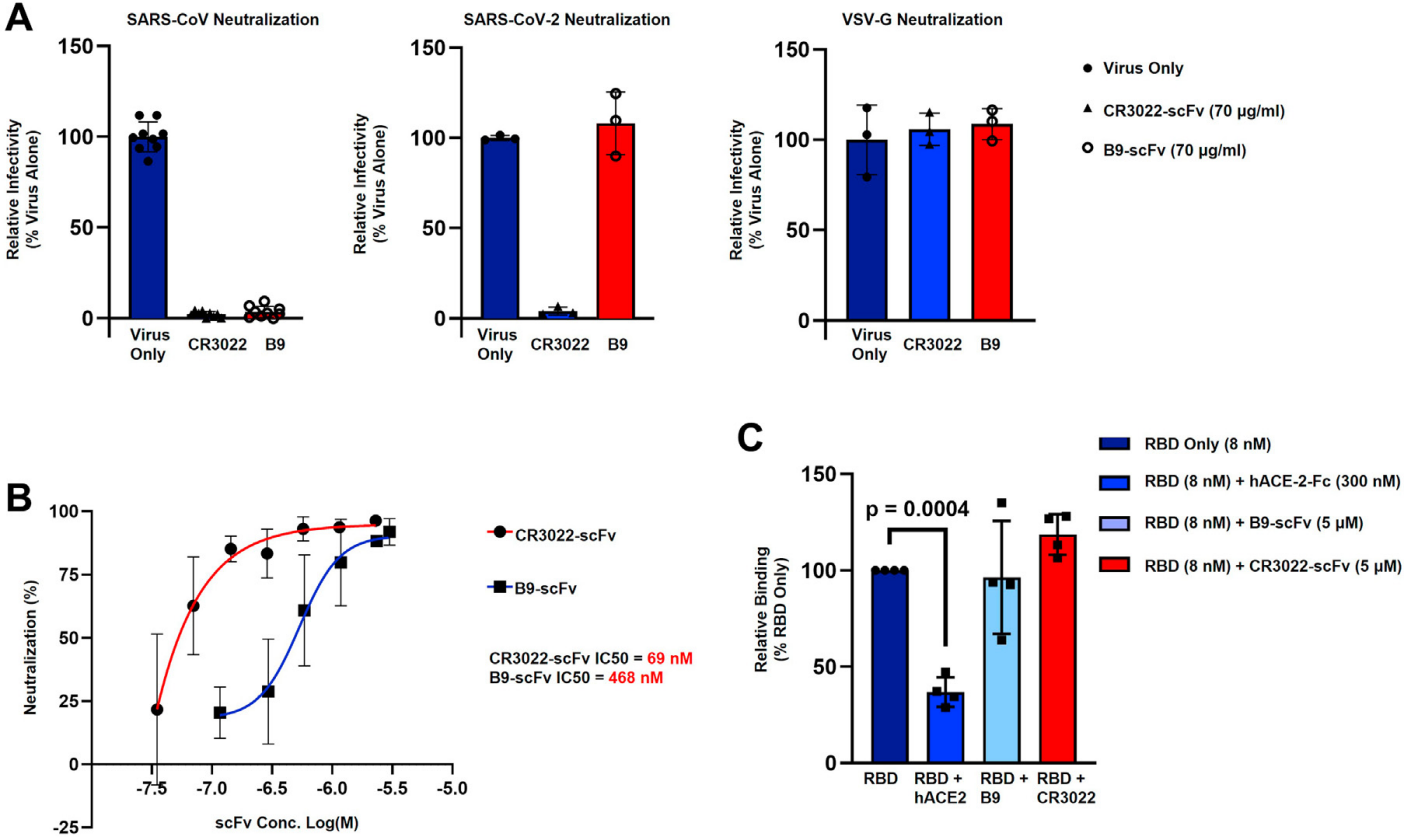
**B9-scFv neutralizes SARS-CoV but does not compete with ACE2.***A*, neutralization of viruses pseudotyped with SARS-CoV Spike protein (*left*; n = 9), SARS-CoV-2 (Wu-1/D614G; *middle*; n = 3), or VSV-G (*right*; n = 3) by the indicated concentrations CR3022-scFv or B9-scFv. Data are presented as mean ± SD. *B*, estimation of the IC_50_ of CR3022- (n = 3) or B9-scFv (n = 4) for the neutralization of SARS-CoV pseudotyped lentiviruses. Data are presented as mean ± SD. Estimated IC_50_ values are shown. *C*, FACS-based quantification of SARS-CoV RBD binding to hACE2 in the presence of the indicated concentrations of ACE2-Fc, B9-scFv, or CR3022-scFv (n = 4). Data are presented as mean ± SD, *p* = 0.0004 (unpaired Student’s *t* test). FACS, fluorescence-activated cell sorting, RBD, receptor-binding domain.

Given that B9-scFv appears to be broadly reactive, based on its resistance to all current SARS-CoV-2 receptor-binding motif mutations and its cross-reactivity with the SARS-CoV RBD (Fig. 3, *B–F*), we next sought to determine if it also neutralizes virus infectivity. We therefore capitalized on the higher affinity of B9-scFv for SARS-CoV Spike to ask whether B9-scFv neutralizes pseudotyped LV particles. As can be seen in Figure 4*A*, B9-scFv almost completely neutralizes lentiviral particles pseudotyped with the SARS-CoV Spike (Urbani variant) when tested at 70 μg/ml (97.9% ± 1.9% neutralization) but has no consistent effect on an equivalent titer of SARS-CoV-2 (Wu-1-D614G) pseudotyped virus, correlating with previously observed differences in estimated affinity (Fig. 3*D*). Control experiments show that B9-scFv does not reduce the infectivity of VSV-G pseudotyped LV at 70 μg/ml (Fig. 4*A*), whereas titration of B9-scFv demonstrates that it neutralizes SARS-CoV pseudotyped LVs with an IC_50_ of 468 nM (Fig. 4*B*).

In complementary experiments, we used B9-scFv in a competition-binding assay to test if it prevents purified SARS-CoV RBD from binding to hACE2-expressing cells. Although there is significant competition between soluble hACE2-Fc (300 nM) and cell surface ACE2 for RBD binding (Fig. 4*C*), high concentrations of B9-scFv (5 μM) or CR3022-scFv (5 μM) did not prevent SARS-CoV RBD from binding to cell surface ACE2. This distinct lack of competition suggests that the mechanism of B9-scFv neutralization is unlikely to involve direct interference with ACE2 binding.

### B9-scFv binds to a cryptic site on the RBD

**Figure 5:**
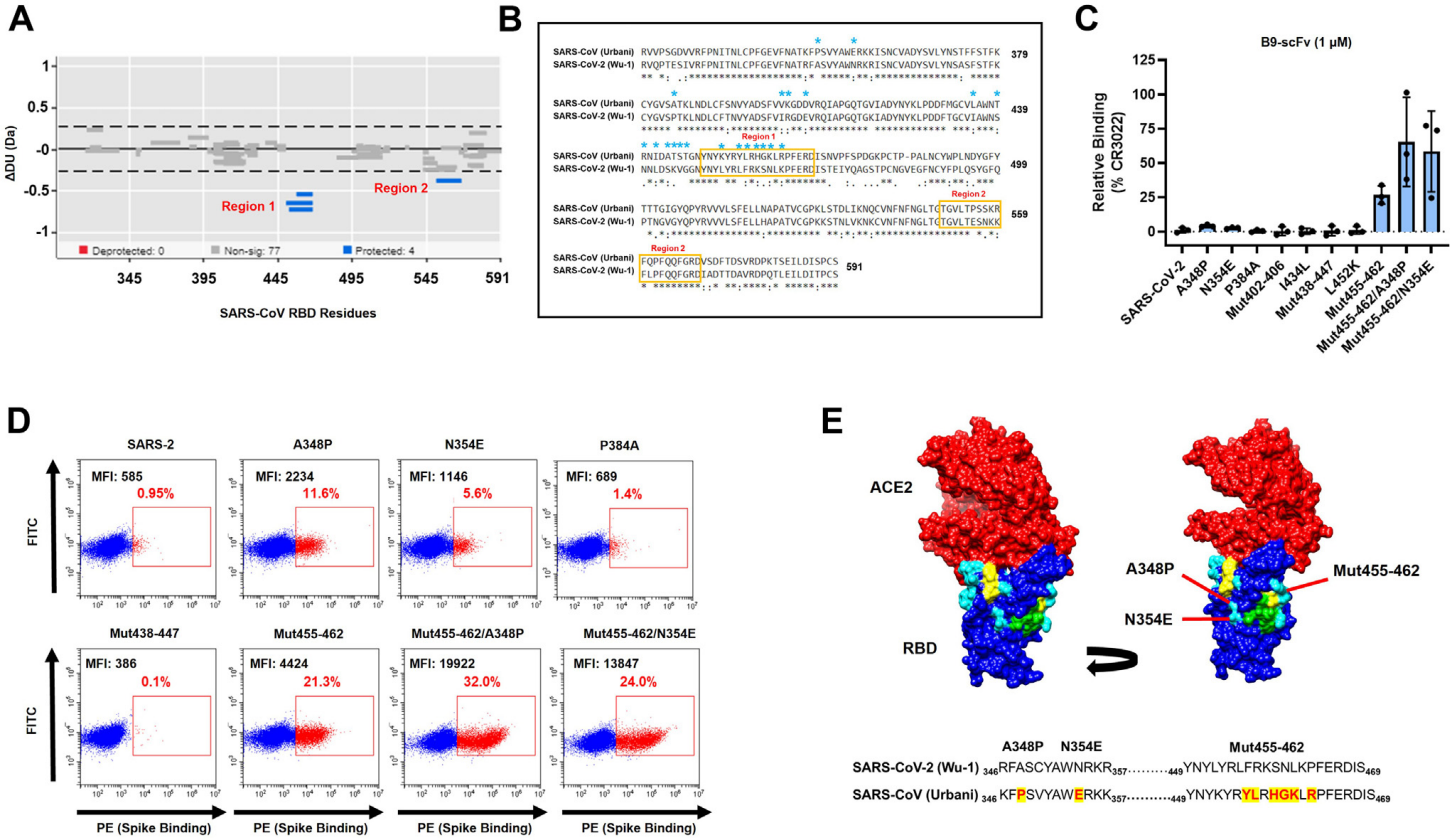
**B9-scFv binds to a conserved, cryptic site on the RBD.***A*, Wood’s plots showing the summed differences in deuterium uptake in SARS-CoV RBD at 2 min of exposure to deuterium, comparing RBD alone to RBD in the presence of B9-scFv. Wood’s plots were generated using Deuteros (46). Peptides colored in *blue* are protected from exchange in the presence of B9-scFv. Peptides with no significant difference between conditions, determined using a 99% confidence interval (*dotted line*), are shown in *gray* (n = 3). *B*, amino acid sequence alignments of SARS-CoV (Urbani) and SARS-CoV-2 (Wu-1) RBDs (aa 319–591). *Orange boxes* around the sequence indicate a protected region on the SARS-CoV RBD when incubated with B9-scFv as identified by HDX. A *cyan asterisk* above the sequence indicates the residues in SARS-CoV-2 that were mutated to their SARS-CoV equivalent in the binding studies shown in (*C*). *C* and *D*, binding of B9-scFv to SARS-CoV-2 (Wu-1) Spike or the SARS-CoV-2 Spike carrying the indicated mutations. Quantification of binding is shown in (*C*; n = 3); selected FACS plots are shown in (*D*). For (*C*), data presented is mean ± SD (n = 3). *E*, modeling the proposed epitope of B9-scFv onto a crystal structure of SARS-CoV-2 RBD (*blue*) bound to ACE2 (*red*) (PDB: 6M0J). HDX protected region 1 (RBD residues 449–467) is in *yellow*, while residues within 5 Å of this are in *green* or *cyan*. Those in *cyan* indicate those mutations tested in (*C* and *D*), arrows indicate the mutations that increase binding. The alignments of the relevant regions of the SARS-CoV and SARS-CoV-2 RBDs are presented beneath the structural models. Mutations that improve binding in (*C* and *D*) are highlighted in *red* and labeled above the sequence. FACS, fluorescence-activated cell sorting, HDX, hydrogen-deuterium exchange; PDB, Protein Data Bank; RBD, receptor-binding domain.

To better localize the epitope recognized by this bovine CDRH3, we took advantage of the higher affinity of B9-scFv for SARS-CoV RBD and the fact that the Spike proteins of SARS-CoV and SARS-CoV-2 are 76% identical (30). We therefore performed differential hydrogen-deuterium exchange mass spectrometry (HDX-MS) of SARS-CoV RBD in the absence (SARS-CoV RBD only) or presence of B9-scFv (SARS-CoV RBD + B9-scFv). HDX-MS is an established methodology for epitope mapping in antibody–antigen complexes (31, 32). In a typical HDX-MS experiment, the exchange of labile amide protons for deuterium is monitored as a function of time. The extent of deuterium incorporation is determined by hydrogen bonding and/or solvent accessibility of the amide protons and is typically measured by MS at the peptide level following proteolysis. The site(s) of difference in deuterium incorporation in the antigen in the absence and presence of the antibody can then be localized, with the regions of protection from deuterium incorporation comprising candidate epitope regions. Here, after a 2 and 30 min exposures to deuterium, we identified two main regions of the SARS-CoV RBD with significantly reduced deuterium uptake in the presence of B9-scFv (Figs. 5*A* and S5). Protected region 1 includes three peptides involving RBD residues 449 to 467 and spans from β7 near the ACE2-interacting region, through the β7-β8 loop to an inner face of the RBD (Fig. S6*B*). Region 1 can be further resolved to just residues 456 to 467 due to the identification of a shorter protected peptide (Fig. S6*A*). In contrast, region 2 comprises only a single peptide of residues 551 to 565 within subdomain 1 and appears to be markedly less protected than region 1 (Fig. 5*A*). Indeed, protection at region 2 was not observed after a 0.5 min incubation with deuterium in the presence of B9-scFv but protection was significant at region 1 at this timepoint (Fig. S5). Given that residues 542 to 591 can be removed with only a small reduction in B9 binding to SARS-CoV RBD (Fig. S6*C*), region 2 does not appear to be the main epitope and we therefore focused on region 1.

Interestingly, region 1 contains a candidate motif, _463_PFERD_467,_ that is fully conserved between SARS-CoV-2 and SARS-CoV and may explain the cross-reactivity of B9-scFv (Fig. 5*B*). To further investigate this, residues surrounding this motif were mutated in full-length SARS-CoV-2 Spike to their equivalents in SARS-CoV (Fig. S6*D*), with the hypothesis that only mutations proximal to the *bona fide* epitope will improve binding. The various SARS-CoV-2 mutants were transfected into 293T cells and subsequently incubated with a concentration of B9-scFv (1 μM) at which binding to SARS-CoV-2 Spike is minimal. Three mutations strengthen the interaction with B9-scFv, namely A348P, N354E, and the patch of mutations, Mut455 to 462 (Fig. 5, *C* and *D*). Of these, Mut455 to 462 has the largest effect and is located adjacent to the _463_PFERD_467_ motif in both the primary amino acid sequence and tertiary structure of the RBD (Fig. 5, *B–E*). Similarly, although the A348P and N354E mutations are distal from _463_PFERD_467_ in the primary amino acid sequence, they are in very close proximity in the tertiary structure (Figs. 5*E* and S6*B*) and are situated on the opposite side of _463_PFERD_467_ relative to Mut455 to 462 (Fig. 5*E*). In contrast, mutations located away from _463_PFERD_467_ on the globular RBD, such as P384A, Mut402 to 406, Mut438 to 447, I434L, and L452K, had no effect on B9-scFv binding to the mutated SARS-CoV-2 Spike (Fig. 5, *C–E*). When combined, the mutations A348P, N354E, and Mut455 to 462 increased binding of B9-scFv to SARS-CoV-2 to levels comparable to the positive control (CR3022-scFv; Fig. 5*C*). The increased strength of B9-scFv’s interaction with SARS-CoV can therefore largely be accounted for by a handful of residues that vary between SARS-CoV-2 (Wu-1-D614G) and SARS-CoV (Urbani). Furthermore, the region mapped by these experiments corresponds to the smaller HDX protected peptide of residues 456 to 467 in region 1 (Fig. S6*A*), supporting the idea that this is the epitope recognized by B9-scFv.

### Potential mechanism of neutralization

**Figure 6:**
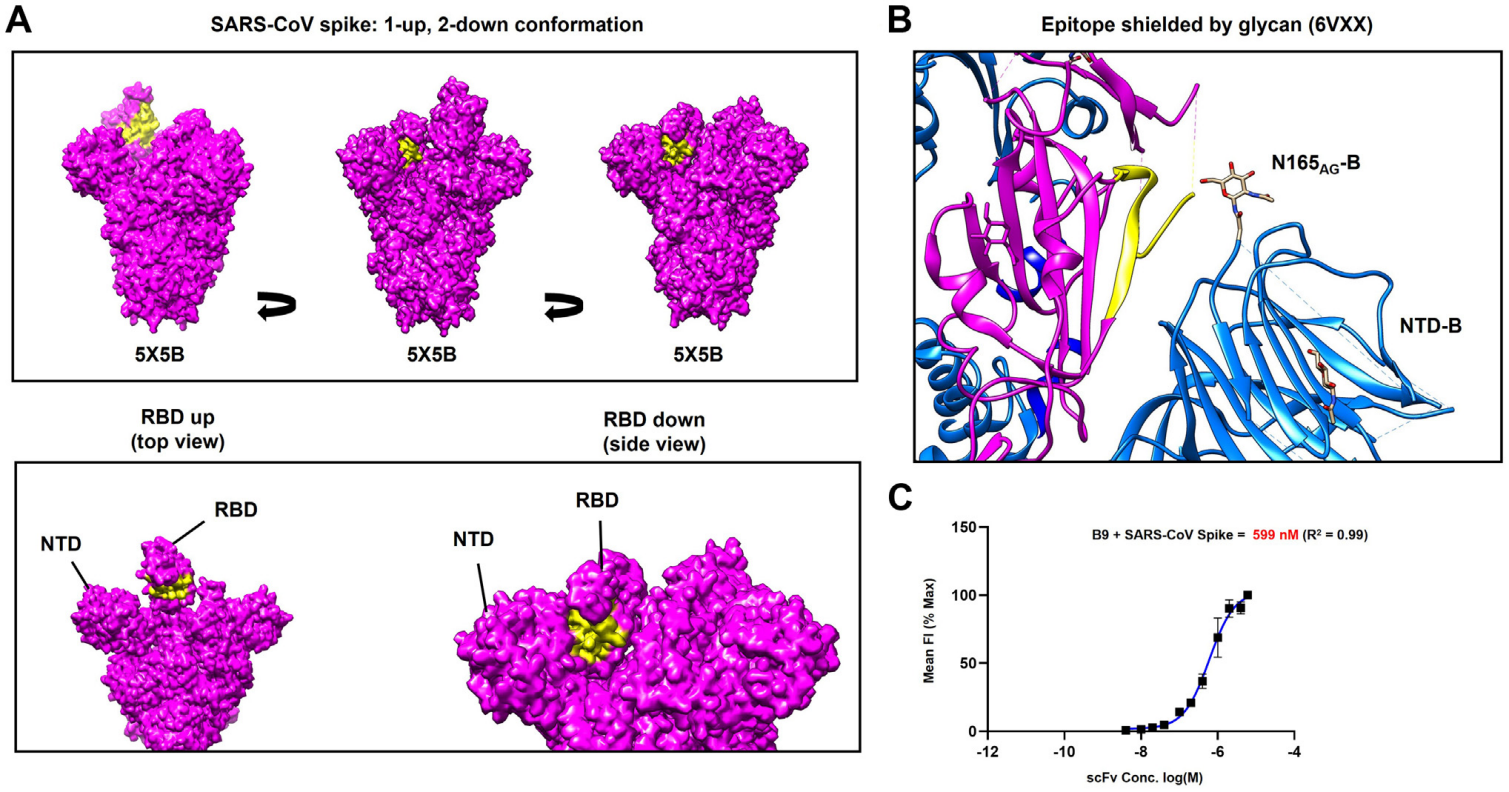
**B9-scFv binding to the cryptic epitope interferes with a stabilizing glycan interaction.***A*, molecular surface map of the full-length SARS-CoV spike (PDB: 5X5B), colored in *magenta*, was generated using UCSF Chimera. The *yellow surface* indicates the proposed epitope of B9-scFv; this region is relatively occluded in all contexts. In an RBD-down conformation, this epitope is pressed against the N-terminal domain of the neighboring protomer, while in the RBD-up conformation it is likely to be inaccessible without extensive clashes. *B*, ribbon diagram showing the epitope for B9-scFv in *yellow* on Spike protomer A (*magenta*). This lies adjacent to the NTD from protomer B (*blue*) where a glycan on N165 potentially occludes conserved epitope. PDB file: 6VXX. *C*, Estimation of the half-maximal binding of B9-scFv to cell surface SARS-CoV Spike (n = 2). NTD, N-terminal domain; PDB, Protein Data Bank; RBD, receptor-binding domain.

Interestingly, in the context of a 1-up, 2-down conformation SARS-CoV Spike trimer, the epitope for B9-scFv is relatively inaccessible on all protomers (Fig. 6*A*). This site has, however, previously been proposed to be transiently exposed by interdomain movements (22). Notably, a glycan from the N-terminal domain (NTD) of the adjacent protomer (N165) is thought to block access to this region by inserting itself in the volume left by the RBD when it is in the “up” conformation (33). This glycan forms the remaining contact between NTD_B_ and RBD_A_ (Fig. 6*B*) to stabilize RBD-up *via* a “load and lock” mechanism (33). Transient domain movements that allow B9-scFv binding would break this glycan contact, potentially destabilizing the Spike complex. Consistent with this, two previous neutralizing antibodies that target this region (7D6/6D6) cause destabilization of the prefusion Spike complex (22) and shedding of the SARS-CoV-2 S1 domain. However, we were unable to detect S1 shedding from the SARS-CoV-2 trimeric Spike following incubation with B9-scFv; this is likely due to the low affinity of B9-scFv for SARS-CoV-2.

Nonetheless, if the epitope is truly occluded, then we would predict that binding of B9 to the SARS-CoV Spike protein will be reduced compared to binding to the RBD alone. We therefore titrated B9 and examined binding to SARS-CoV Spike expressing cells. Consistent with our idea, half-maximal binding was detected at 599 nM (Fig. 6*C*), which is 3-fold higher than the *K*_*D*_ for the interaction of B9 with SARS-CoV RBD.

Finally, bovine bNAbs normally bind their epitopes *via* the CDRH3 knob domain, and to test if this is the case for B9-scFv, we disrupted the knob domain by mutagenesis. Three consecutive regions of the knob domain were replaced with the irrelevant amino acid sequence (B9Mut1-3, ETYYGSGL; 15). As can be seen in Fig. S7, mutations at the N terminus of the knob domain (B9Mut1 & B9Mut2) have a marked impact on both scFv cell surface expression and RBD binding, suggesting that they likely impact on the folding or overall stability of the ultralong CDRH3. In contrast, mutation at the C terminus (B9Mut3) has little impact on scFv expression but abrogates RBD binding, indicating that this region may include critical paratope residues. Consistent with this idea, mutation of just four amino acids within this C-terminal region (WYRY; B9Mut4) to alanine is sufficient to completely abolish the B9-scFv/RBD interaction across a wide range of RBD concentrations (Fig. S7*D*). Together, these data provide strong evidence that B9-scFv uses its ultralong knob domain to engage a site largely comprised of conserved residues in the β7-β8 loop of the *Sarbecovirus* Spike RBD to trigger neutralization.

## Discussion

The increasing frequency of zoonotic transfers, together with the possibility of rapid antigen evolution, highlights the urgent need for broadly active therapeutics to fight emerging pathogens. Using our novel scFv display system to specifically express bovine ultralong heavy chains in mammalian cells, we have isolated a bovine ultralong scFv (B9) that targets the RBD of the SARS-CoV-2 and SARS-CoV Spikes, as well as all SARS-CoV-2 variants, at a conserved but cryptic site. nAbs targeting this region (Fig. S6*B*) are associated with exceptional breadth (7, 22, 23) and deep mutational scanning previously suggested this site represented the “ideal” epitope (24) as the most conserved region of vulnerability within the RBD (23, 24). Notably, antibodies that were previously shown to target this site, 7D6/6D6, were isolated only after five immunizations of mice with either SARS-CoV-2 S-2P or a combination of SARS-CoV-2 Spike, SARS-CoV Spike, and MERS-RBD; by contrast, our present study isolated B9-scFv from a small, naïve library of bovine CDRH3s. In fact, the identification of an ultralong CDRH3 targeting a seemingly rare neutralizing epitope is made even more remarkable given the modest size of our initial library. This study therefore demonstrates the power of bovine ultralong CDRH3 regions to identify highly conserved sites of vulnerability on viral antigens. We further suggest that our system provides an efficient new tool to identify broadly reactive paratopes by affinity-based panning and, furthermore, that with an expanded library, the likelihood of identifying higher affinity binders to SARS-CoV-2 and other potentially pathogenic *Sarbecoviruses*, will be substantially increased.

A subset of bovine ultralong antibodies possess the longest known CDRH3 regions. It is likely that the extended β-stranded stalk helps to penetrate through glycan coats on some viral antigens to reach occluded, functionally conserved epitopes, while it has been shown that the disulfide-bonded loops can engage a target using a compact surface area. Together, these structural features increase the resistance of ultralong nAbs to viral escape mutations (17). In keeping with previously studied antibodies with an ultralong CDRH3, B9 engages its glycan-shielded epitope with knob domain-only binding since disruption of the knob by mutation completely abrogates its interaction with SARS-CoV RBD (Fig. S7). Unusually, however, B9-scFv has a truncated ascending and descending β-stranded stalk (15, 21), with fewer residues at the VD junction (_101_RD_102_) and no alternating tyrosine motif at the 3′ end of D_H8-2_. These features will likely impact the length, angle, and flexibility of the stalk and may influence how the disulfide-bonded loops of the B9 knob domain engage the RBD.

Notably, when the epitope for B9-scFv is mapped onto a model of a trimeric SARS-CoV Spike in the 1-up, 2-down RBD conformation, it appears relatively inaccessible in every context. Our HDX-MS and mutagenesis data show B9-scFv’s epitope appears to share several key contacts on the β7-β8 loop of the RBD with previously identified cross-nAbs, including 7D6 (22), FD20 (23) and S2H97 (7). The binding of B9-scFv is therefore also likely to clash with the NTD from the adjacent protomer in the context of the trimeric Spike (Fig. 6*B*). The adjacent NTD contains a crucial aminoglycan moiety at N165 that has been shown to be critical for gating of RBD opening and may also act to shield this conserved region from antibody recognition (Fig. 6*B*; (22, 33)). Indeed, previous models suggest this cryptic epitope is only made accessible by transient movements of the RBD and NTD, and subsequent antibody binding acts to destabilize the trimer. This ultimately leads to neutralization by the destruction of the prefusion SARS-CoV and SARS-CoV-2 Spikes and shedding of the S1 domain in the latter (22). Interestingly, B9 appears to interact less well with full-length Spike than RBD only (Figs. 3*E* and 6*C*). This effect may be the result of steric hindrance caused by the aminoglycan at N165 and was also observed for 7D6 (22). Moreover, B9-scFv binding to this cryptic epitope correlates with its relative cross-reactivity, lack of competition with ACE2, and resistance to receptor-binding motif mutations found in SARS-CoV-2 VOC.

Despite being isolated from a relatively small, naïve library, the ability of B9-scFv to neutralize SARS-CoV was only moderately less than antibodies isolated from convalescent patients that were subsequently developed as therapeutics (28). It may be possible to improve the binding of isolated bovine scFvs to generate pathogen-specific therapeutic tools, either by directing activation-induced cytidine deaminase–mediated mutagenesis, using CRISPR-x (34), or through other diversification methods, such as error-prone PCR or structure-guided affinity maturation (35). Indeed, all mutagenesis efforts could be entirely focused on the region encoding the knob domain. It is also probable that with larger initial libraries, binders against more diverse and cross-reactive epitopes will be isolated. At least some of these would be expected to have a high initial affinity and require fewer mutations, if any, to achieve strong target binding. Previous efforts to select and engineer human antibodies for increased breadth and potency against SARS-like viruses have been remarkably successful (36). It should be noted, however, that bovine ultralong CDRH3s are unlikely to be amenable to typical oligonucleotide-based CDR diversification methods, due to the length and unique structural requirements of the ultralong CDRH3 (15, 21).

Clearly, identifying a novel paratope is just the first step in developing a treatment against a new pathogen, as a nonimmunogenic scaffold with which to deliver the neutralizing agent is crucial. Fortunately, various possibilities exist. Firstly, bovine paratopes have been successfully transferred to a human antibody scaffold with minimal loss of activity (16), although optimization of a scaffold may be required to achieve stability and good manufacturability (19). Other options include scFv-Fc fusions (37) or PEGylated scFvs (38), both of which have significantly improved pharmacokinetic profiles over standard scFvs. Nonetheless, a risk remains that the bovine ultralong CDRH3s will be immunogenic when administered intravenously. This has not proven to be a problem for llama paratopes but these CDRH3s are considerably shorter (median 16 aa *versus* >50 aa) than their bovine counterparts. If this does prove to be an issue for the ultralong bovine CDRH3s, it may be feasible to nebulize modified bovine scFvs or smaller neutralizing fragments such as knob peptides (39), for delivery to the sites of virus entry (40), as has been suggested for heavy chain–only (V_H_H) nanobodies.

Pandemic preparedness and responsiveness hinges on the rapid identification of neutralizing epitopes. The emergence of three beta-coronaviruses of pandemic potential in the last 20 years indicates the huge risk posed by these viruses and their high incidence of zoonotic transfers. Indeed, with the substantial CoV diversity in bats alone (41), SARS-CoV-2 will not be the last emergence. Therefore, the generation of libraries of bNAbs that recognize this virus group will allow rapid screening against emerging related viruses, and as exemplified by the studies here, even if an antibody has a relatively weak affinity to one virus subtype, it may have a substantially higher affinity for a related pathogen. Therefore, with a significantly expanded ultralong CDRH3 library, we speculate that our pipeline would be able to identify ultralong nAbs that can be rapidly deployed against new pathogens, without the need for animal immunizations. Not only this, but the epitopes identified by these bNAbs may provide targets for more durable vaccines that confer better protection against new variants.

## Experimental procedures

### Mammalian cell culture

293T cells were grown in Dulbecco’s modified Eagle's medium (DMEM) supplemented with 10% fetal calf serum, 4 mM L-glutamine, 50 U/ml penicillin, and 50 μg/ml streptomycin. Cells were grown in a humified incubator at 37 °C with 5% CO_2_.

### Generation of an ultralong scFv library for mammalian cell surface display

To generate the pBovShow expression vector, DNA encoding an scFv expression cassette, with Ig kappa (*IGK*) leader sequence, (GGGS)_x3_ linker, Myc epitope tag, and platelet-derived growth factor receptor transmembrane domain (PDGFR-TM), was synthesized by IDT and cloned into the EcoRV and XbaI sites of the pCS2-MT+ vector (Addgene plasmid #2296). To generate bovine variable domains, we first isolated gDNA from the leucocytes of two heifers. The first animal was a 21-month-old British Blue (Shorthorn x Friesian), while the second was a 23-month-old Limousin x Holstein. Both animals were raised in Northern England where typical vaccinations include those against bovine viral diarrhea, bovine respiratory syncytial virus, infectious bronchial rhinitis, and an anti-Clostridium vaccine that protects against 10 different *Clostridium* strains. Sequences encoding V_λ_-light chain variable domains (LCs) were amplified from these bovine leukocyte gDNA samples using the primers described (42). Individual clones were Sanger sequenced and screened for homology to V_λ_-LCs that are known to productively pair with ultralong heavy chains (42). A sequence with 99% homology to the BLV1H12 V_λ_-LC was cloned into the XhoI and XmaI sites in the scFv expression cassette, downstream of the (GGGS)_x3_ linker. An ultralong heavy chain library was generated using nested PCR and the same bovine gDNA samples. First round amplification was performed with a forward primer hybridizing to a unique region upstream of V_1-7_ (5′-GGACCCTCCTCTTTGTGCTCTCAG-3′), whereas the second round forward primer was V_1_-specific (5′-TCACGCTAGCCAGGTGCAGCTGCGGGAGTCG-3′; both PCRs used a J_2-4_ specific reverse primer (5′-GGATAGATCTCTGAGGAGACGGTGACCAGGAG-3′). The final amplicons, flanked by XbaI and BglII sites, were gel purified and cloned into our display vector upstream of the (GGGS)_x3_ linker. A table showing the nucleotide sequences of the B9 heavy chain, VL variable domain, and the expression cassette depicted in Fig. 1*B* is given (Table S1). The entire ligation reaction was transformed into DH5α competent *Escherichia coli* cells and used to inoculate an overnight midi culture for preparation of polyclonal plasmid DNA encoding the scFv library.

### Purification of His-tagged proteins by immobilized metal affinity chromatography

Full-length trimeric Spike protein, residues 1 to 1208, was kindly provided by the Oxford protein production facility. It has proline substitutions at residues 986 and 987, a GSAS substitution at the furin cleavage site (residues 682–685), a C-terminal T4 fibritin trimerization motif, an HRV3C protease cleavage site, a TwinStrepTag, and an 8xHisTag. It was expressed in mammalian FreeStyle293F cells and purified *via* IMAC. An expression vector for the secretion and purification of His-tagged proteins from mammalian cells was generated by insertion of DNA encoding an IGK leader and 8xHis tag into pCS2-MT+. A DNA fragment encoding the SARS-CoV-2 RBD (aa 319–591) was amplified from pCAGGS-SARS-CoV-2-Spike vector (a kind gift from Dr Keith Grehan) and cloned in frame with the N-terminal IGK leader sequence and C-terminal 8xHis tag using NheI and XhoI sites. DNA fragments encoding the SARS-CoV RBD (aa 319–591) and MERS-CoV RBD (aa 368–586) were synthesized by IDT and cloned into the vector using the same restriction sites. All DNA sequences encoding scFvs for purification were subcloned from the mammalian display vector into this purification vector using EcoRV and XhoI sites. Proteins were recovered from the supernatants of 293T cultures following transient transfection. Briefly, 5 × 10^6^ 293T cells were transfected with 15 μg of relevant protein expression plasmid in 15 cm^2^ dishes using PEI at a 1:3 DNA:PEI ratio. Complete media was replaced with serum-free media 24 h after transfection. After a further 96 h, the supernatant was collected and cleared by centrifugation at 4000*g* for 5 min before being filtered through a 0.45 μm syringe filter (Fisher). Imidazole (Merck) was added to the cleared supernatants to a final concentration of 10 mM and the supernatants were incubated with 1 ml nickel resin (Generon; 50% slurry equilibrated in PBS) on a roller at 4 °C for 30 min to bind His-tagged proteins. The resin was loaded onto a 20 ml econo-column (Bio-Rad) and extensively washed with increasing concentrations of imidazole in PBS (10, 20, and 30 mM). Bound proteins were eluted with 250 mM imidazole in PBS. Protein containing fractions were determined by A^280^ measurements and SDS-PAGE. The required fractions were extensively buffer exchanged into PBS + 10% glycerol using a 10 kDa MWCO centrifugal filter (Millipore) and concentrated to >1 mg/ml for storage at −80 °C.

### Flow cytometry analysis of cell surface displayed scFv interactions

A standardized staining protocol was followed prior to fluorescence analysis on a Cytoflex S cell analyzer (Beckman) and FACS on a FACSMelody (Becton Dickinson; BD). Briefly, 293T cells expressing scFv on the cell surface were detached with trypsin-EDTA (Thermo Fisher Scientific) and washed twice in prechilled sort buffer (1% fetal calf serum, 25 mM Hepes–KOH pH 7.9, 1 mM EDTA in PBS). Cells were resuspended in sort buffer to 1 × 10^7^/ml and incubated with the indicated concentrations of target His-tagged proteins for 1 h at 4 °C. After binding, samples were washed twice in sort buffer and incubated with a 1:100 dilution of α-Myc-FITC (Abcam, #Ab1263) and α-His-PE (Abcam, #Ab72467) antibody at room temperature (RT) for 10 min. Following staining, cells were washed twice in sort buffer and resuspended at 1 × 10^6^/ml for flow cytometry.

### Plasmid recovery

Cells expressing Spike-binding scFvs were purified by flow cytometry, followed by centrifugation at 600*g* for 3 min. The respective plasmid expression vectors were recovered by resuspending the pelleted cells in 100 μl of Hirt I solution (0.6% SDS, 10 mM Tris–HCl pH 8.0, 1 mM EDTA; (43)) and incubation at RT for 10 min. Next, 50 μl of Hirt II solution was added (5 M NaCl, 10 mM Tris–HCl pH 8.0, 1 mM EDTA), lysates were mixed, and incubated at 4 °C overnight. Lysates were centrifuged at 16,000*g* for 40 min and plasmid DNA was recovered by phenol:chloroform extraction of the supernatant, followed by ethanol precipitation and resuspension in 10 μl of ddH_2_O. DH5α competent *E. coli* (High Efficiency; NEB) were chemically transformed with 5 μl of the recovered plasmid library and incubated for 1 h at 37 °C with shaking at 220 rpm. This was used to inoculate an overnight culture for midi-scale preparation of plasmid scFv library DNA. Three rounds of plasmid-based selection were performed and at each stage Spike binding was verified by transient transfection, while the sequences recovered were characterized by amplicon sequencing.

### Amplicon sequencing and library size estimation

The scFv library was subjected to amplicon sequencing following rounds 0, 2, and 3 of the plasmid-based enrichment. Briefly, 10 μg of plasmid scFv library from the relevant round of enrichment was digested with EcoRV and BglII to yield a 500 bp fragment spanning the entire ultralong V_H_ sequence. The DNA fragment was gel purified and its concentration adjusted to 20 ng/μl. The resulting fragment library was sequenced by the Illumina-based Genewiz, Amplicon-EZ service to generate 2 × 250 bp paired-end reads. Only reverse reads were used for analysis as they span the whole CDRH3 region and allow accurate characterization of unique CDRH3 sequences. Raw FastQ files from Genewiz were filtered for quality and converted to Fasta format. Mixcr ((44) https://github.com/milaboratory/mixcr) was used to align the reads to bovine V, D, and J segments using *Bos taurus* IMGT libraries for assignment. Unique clonotypes were then assembled and ranked by proportion. The number of unique clonotypes in each case was eventually used to approximate the initial library diversity using the capture-mark-recapture formula $N=\frac{Mn}{m}$, where N = heavy chain library size to be estimated, M = unique heavy chain sequences recovered in round 0, n = unique heavy chain sequences recovered in round 2 or round 3, and m = sequences found both in round 0 and round 2 or 3.

### LV particle generation, transduction, and scFv isolation

A lentiviral plasmid for the stable expression of ultralong scFvs was generated by modifying LentiCRISPR v2 (Addgene plasmid #52961, a gift from Feng Zhang). An internal ribosomal entry site (IRES) was cloned downstream of the PDGFR-TM sequence in pBovShow and the whole cassette was inserted between the EF1α core promoter and the puromycin resistance gene of LentiCRISPR v2 to generate Lenti-BovShow-IRES-PuroR. The round 3 enriched ultralong scFv library was transferred from pBovShow into this lentiviral vector and LVs were generated by transient transfection of 293T cells. Briefly, 293T cells were seeded at 3 × 10^6^ cells per 10 cm^2^ dish. The next day, 4 μg Lenti-BovShow-IRES-PuroR, 4 μg of pCMVR8.74 packaging vector (Addgene plasmid #22036), and 2 μg of pMD2.G coat protein vector (Addgene plasmid #12259; both gifts from Didier Trono) were mixed with PEI at a 1:3 M ratio and added to the 10 cm^2^ dish. The medium was changed after 24 h and LV-containing supernatants were collected at 48 and 72 h post-transfection. Transduction of 293T cells was achieved by seeding the cells at 30% to 40% confluency in a 75 cm^2^ flask. The following day, medium was replaced with 9 ml of complete DMEM, plus 1 ml of lentiviral supernatant and polybrene at a final concentration of 5 μg/ml. Medium was replaced after 48 h with complete medium containing 2 μg/ml puromycin dihydrochloride (Cayman Chemical, Item No. 13884). After 1 week, puromycin-selected cells were incubated with SARS-CoV-2 Spike (40 nM) and stained with α-Myc-FITC and α-His-PE antibodies as described. Single cells were purified from the population of Spike-binding cells by flow cytometry using a FACSMelody (BD). Single cell clones were then assessed for binding to His-tagged SARS-CoV-2 Spike using a Cytoflex S (Beckman) cell analyzer. The scFv sequences were amplified from 100 ng of gDNA using a forward primer hybridizing to the scFv-leader sequence (5′- GACTTTGATATCATGGAGACAGACACACTCCTG-3′), a J_2-4_ reverse primer 5’-(GGATAGATCTCTGAGGAGACGGTGACCAGGAG-3′), and Herculase II polymerase (Agilent), following the manufacturer’s recommended reaction conditions. Amplicons were analyzed by Sanger sequencing and cloned into pBovShow expression and purification vectors.

### scFv binding to Spike variants expressed on the cell surface

293T cells were plated at 0.2 × 10^6^ in 6-well plates 24 h prior to transfection. The next day, 1 μg of plasmid vector encoding full-length SARS-CoV Spike (pCAGGS-SARS-CoV-Spike_Urbani), SARS-CoV-2 Spike (NR-52514 SARS-CoV-2 Spike glycoprotein), or SARS-CoV-2 variant Spike was transfected at a 1:3 DNA to PEI ratio. After 48 h, cells were detached, washed twice in sort buffer, and incubated with B9-scFv-8xHis at the concentrations indicated. After 1 h, cells were washed twice in sort buffer, incubated with a 1:100 dilution of α-His-PE antibody (Abcam, #Ab72467) for 10 min, and washed twice more. Stained cells were resuspended in sort buffer at 1 × 10^6^/ml for fluorescence analysis on a Cytoflex S cell analyzer (Beckman). SARS-CoV-2 Spike variants were generated by site-directed mutagenesis with Q5 polymerase (New England Biolabs) and confirmed by Sanger sequencing. Spike cell surface expressions were confirmed by staining with 300 nM of a positive control scFv (CR3022-scFv) that was purified by IMAC (Fig. S4*A*).

### Pseudotype neutralization assays

Pseudotyped lentiviral particles were generated by transfecting 3 × 10^6^ 293T cells in a 10 cm^2^ dish with a LV backbone plasmid encoding a luciferase reporter gene (BEI: NR-52516 pHAGE-CMV-Luc2-IRES-ZsGreen-W), a plasmid encoding either the SARS-CoV Spike (pCAGGS-SARS-CoV-Spike_Urbani), SARS-CoV-2 Spike (BEI: NR-52514), or VSV glycoprotein (VSV-G; pMD2.G) and the packaging vectors HDM-Hgpm2 (BEI: NR-52517), HDM-tat1b (BEI: NR-52518), and pRC-CMV-Rev1b (BEI: NR-52519). DNA was transfected at a 1:3 ratio with PEI. Virus particles were collected 48 and 72 h post-transfection and resultant LVs serially diluted 1:3 (SARS-CoV and SARS-CoV-2) or 1:10 (VSV-G) on 2.5 × 10^5^ hACE2 expressing 293T cells. In each case, the transduction units per ml (TU/ml) was calculated from the percentage of green cells 48 h post-transduction, with TU/ml = (number of cells transduced × % positive)/dilution factor. The TU/ml value was calculated from samples with 1% to 10% green cells. For neutralization experiments, equal titers of pseudotyped LVs were incubated with different concentrations of scFv or hACE2-Fc for 1 h at 37 °C and added to 1.25 × 10^4^ ACE2-expressing cells. After 48 h, the luciferase activity of the infected cells was measured and plotted as a percentage of the virus-only infectivity to generate relative values. All procedures were performed as described by Crawford *et al.* (45), using reagents kindly supplied by BEI, except where indicated. For IC_50_ calculations, SARS-CoV pseudotyped LVs were incubated with 0.02 to 2 μM CR3022- or 0.04 to 3 μM B9-scFvs and used to transduce 293T-hACE2 cells. The IC_50_ of the scFvs was approximated from the nonlinear regression of the summarized log(scFv concentration)-response plots on GraphPad (GraphPad Software Inc).

### ACE2 competition assay

Initially, His-tagged SARS-CoV RBD (8 nM) was incubated with either ACE2-Fc (300 nM), B9-scFv (5 μM), or CR3022-scFv (5 μM) at 4 °C for 30 min in sort buffer. Next, aliquots of 0.25 × 10^6^ HEK-293T-hACE2 (BEI: NR-52511) cells were washed in sort buffer and resuspended in one of the preincubated RBD samples. After 1 h at 4 °C, cells were washed twice in sort buffer and stained with α-His-PE antibody (1:100). After a final two washes in sort buffer, fluorescence was measured by flow cytometry on a Cytoflex S (Beckman). ACE2 competition was assessed by the reduction in RBD binding to the HEK-293T-hACE2 cell surface as a percentage of RBD only.

### SPR

The kinetics of the interaction between B9-scFv and the RBD from SARAS-CoV and SARS-CoV-2 were analyzed by SPR on a Biacore 3000 (Cytiva) at 25 °C. Biotinylated proteins were generated by reacting with NHS-PEG_4_-biotin (Thermo Scientific) at a 1:1 M ratio with desalting to remove unreacted reagent. Proteins were immobilized on a streptavidin sensor chip (Sensor Chip SA, Cytiva) to a low density, and an untreated flow cell was used as a reference surface. The running buffer was PBS with Tween 20 (0.05% v/v). Analyte dilutions were injected for 180 s at 30 μl/min with a dissociation phase of 600 s. The process was performed three times for each analyte. Response from the reference surface and a buffer injection over derivatized surfaces was subtracted from derivatized flow-cell data. Affinity and kinetic rate constants were determined for a 1:1 Langmuir binding model (BIA evaluation v4.1.1).

### HDX-MS

For HDX-MS experiments, a robot for automated HDX (LEAP Technologies) was coupled to an Acquity M-Class liquid chromatography system and HDX manager (Waters). Samples comprised protein (SARS-CoV RBD or SARS-CoV RBD and B9-scFv, at a concentration of 10 μM and 50 μM, respectively) in 50 mM potassium phosphate, pH 8, 0.3 M NaCl. To initiate the HDX experiment, 95 μl of deuterated buffer (50 mM potassium phosphate, pD 8, 0.3 M NaCl) was added to 5 μl of protein-containing solution, and the mixture was incubated at 4 °C for 0.5, 2, and 30 min. For each time point and condition, three replicate measurements were performed. The HDX reaction was quenched by adding 100 μl of quench buffer (10 mM potassium phosphate, 0.05 % n-dodecyl-β-D-maltoside, pH 2.2) to 50 μl of the labeling reaction.

The quenched sample (50 μl) was proteolyzed by flowing through immobilized pepsin and aspergillopepsin columns (Affipro) connected in series (20 °C). The resulting peptides were trapped on a VanGuard Precolumn [Acquity UPLC BEH C18 (1.7 μm, 2.1 mm × 5 mm, Waters)] for 3 min. The peptides were separated using a C18 column (75 μm × 150 mm, Waters) by gradient elution of 0% to 40% (v/v) buffer B in buffer A where buffer A is H_2_O, 0.3% v/v formic acid and buffer B is acetonitrile, 0.1% v/v formic acid over 7 min at 40 μl min^−1^.

Peptides were detected using a Synapt G2Si mass spectrometer (Waters) operating in HDMS^E^ mode, with dynamic range extension enabled. Ion mobility separation was used to separate peptides prior to collision-induced dissociation fragmentation in the transfer cell. Collision-induced dissociation data were used for peptide identification, and uptake quantification was performed at the peptide level. Data were analyzed using ProteinLynx Global Server (PLGS) (v3.0.2) and DynamX (v3.0.0) software (Waters). Search parameters in PLGS were as follows: peptide and fragment tolerances = automatic, minimum fragment ion matches = 1, digest reagent = nonspecific, false discovery rate = 4. Restrictions for peptides in DynamX were as follows: minimum intensity = 1000, minimum products per amino acid = 0.3, max sequence length = 25, max ppm error = 5, file threshold = 3. The software Deuteros (https://github.com/andymlau/Deuteros_2.0) (46) was used to identify peptides with statistically significant increases/decreases in deuterium uptake and to prepare Wood’s plots. The raw HDX-MS data have been deposited to the ProteomeXchange Consortium *via* the PRIDE partner repository with the dataset PXD032965. A summary of the HDX-MS data, as recommended by reported guidelines is shown in Table S2.

### Disruption of the B9-scFv knob domain

The sequence encoding B9-scFv was mutated in the BovShow cell surface expression vector. First, residues 106 to 113, 115 to 123, and 123 to 131 of the B9-scFv knob domain (B9-WT) were replaced with the irrelevant amino acid sequence ETCYYGSGL by site-directed mutagenesis to generate three B9 mutants (B9Mut1-3). In subsequent experiments, residues 130 to 133 were replaced with AAAA to generate B9Mut4. All mutagenesis was performed using Q5 polymerase (New England Biolabs) and codons encoding cysteine residues were left unmutated. 293T cells were transfected with either B9-WT or the indicated B9 mutant plasmid DNA and cell surface–expressed scFvs were tested for binding to purified SARS-CoV RBD (200 nM) using the standard staining protocol.

### Structural models used in this study

The Protein Data Bank files used in this study were 4K3D, 6M0J, 5X5B, and 6VXX. All structural figures used in this study were generated in UCSF Chimera.

### Statistical information

Flow cytometry experiments include a positive and unstained negative control and were performed at least in triplicate and/or with sufficient replicates to ensure statistically significant data (except Figs. 3*E* and 6*C* that were performed in duplicate). Quantification of binding is determined using mean fluorescence intensity *via* CytExpert2.4 and is plotted to show the mean ± SD. The *K*_*D*_ for interactions between cell surface scFvs and recombinant RBD proteins was estimated by nonlinear analyses of the log(molarity)-response plots on GraphPad.

Pseudotype neutralization assays were performed at least in triplicate to calculate the SD of percentage neutralization (compared to the negative control) at each concentration of scFv. IC_50_ values are calculated from the nonlinear regression of log(molarity) of scFv *versus* percentage neutralization.

For differential HDX-MS, peptide-level significance testing was implemented in Deuteros 2.0 (46) to identify peptides with significant differences in deuterium uptake in the bound state. A hybrid significance test was used that first evaluates if the difference in deuterium uptake between two states is greater than a threshold value that corresponds to a significance level of *p* < 0.01. This was followed by a Welch’s *t* test to confirm that the differences are significant.

## Data availability

The dataset that was generated in this study is available in the following database:

Protein interaction HDX-MS data:

PRIDE PXD032965 (http://www.ebi.ac.uk/pride/archive/projects/PXD032965)

## Supporting information

This article contains supporting information.

## Conflicts of interest

The authors declare that they have no conflicts of interest with the contents of this article.
