# Supplementary material for: A bovine antibody possessing an ultralong complementarity-determining region CDRH3 targets a highly conserved epitope in sarbecovirus spike proteins
Source: J Biol Chem. 2022 Oct 20;298(12):102624. doi: 10.1016/j.jbc.2022.102624 (PMC9678781; doi:10.1016/j.jbc.2022.102624)
Supplement: Supporting information [file mmc1.pdf]

A bovine antibody possessing an ultralong complementarity-determining region CDRH3 targets a highly conserved epitope in *sarbecovirus* spike proteins

Matthew J. Burke, James N.F. Scott, Thomas C. Minshull, Zeqian Gao, Iain Manfield, Sinisa Savic, Peter G. Stockley, Antonio N. Calabrese and Joan Boyes

Figure S1. Estimation of scFv library diversity.

Figure S2. Lentivirus transduction of scFv library.

Figure S3. Isolation of an ultralong scFv that binds to the wildtype SARS-CoV-2 Spike and receptor binding domain.

Figure S4. Purified B9-scFv binds to SARS-CoV-2 Spike expressed on the surface of 293T cells.

Figure S5. Hydrogen-deuterium exchange experiments for epitope localisation.

Figure S6. Localising the epitope of B9-scFv to a cryptic site by hydrogen-deuterium exchange and site-directed mutagenesis.

Figure S7. Mutagenesis of the B9 knob domain.

Table S1: Nucleotide sequences in the expression cassette.

Table S2: HDX Data Summary Table.

A

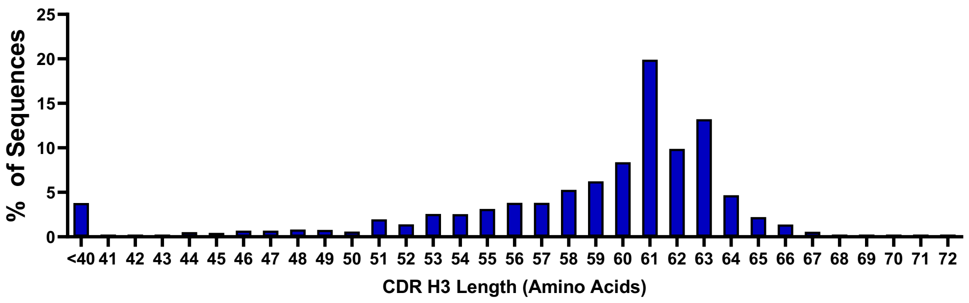

B

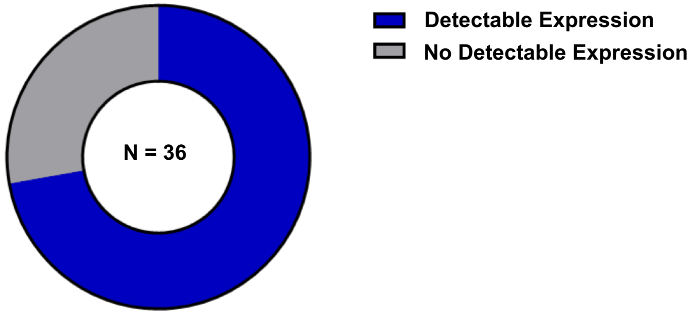

C

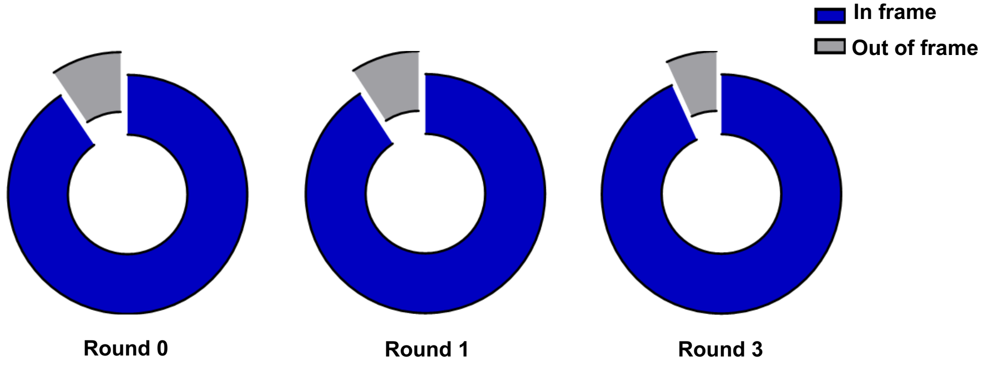

	Round 0	Round 2	Round 3
**Unique**	3168	379	250
**Non-unique**	N/A	260	102

**Estimated Library Size**	
**Round 2**	**Round 3**
4617	7764

Fig. S1

Figure S1. Estimation of scFv library diversity. *A*, Histogram showing the proportion of heavy chain amino acid sequences with CDRH3 between 40 and 72 amino acids. Heavy chain amino acid sequences were determined from the amplicon sequencing of the Round 0 plasmid scFv library. *B*, Random pBovShow-scFv clones were (n = 36) transfected into 293Ts and tested for cell-surface expression. The positive fraction is indicated in blue. *C*, Clonotypes were assembled from amplicon sequencing of the scFv plasmid library at three different time points during the Spike-binding enrichment (Round 0, Round 2 and Round 3). The resultant clonotype assemblies were used to assess the fraction of productive and non-productive heavy chain sequences.

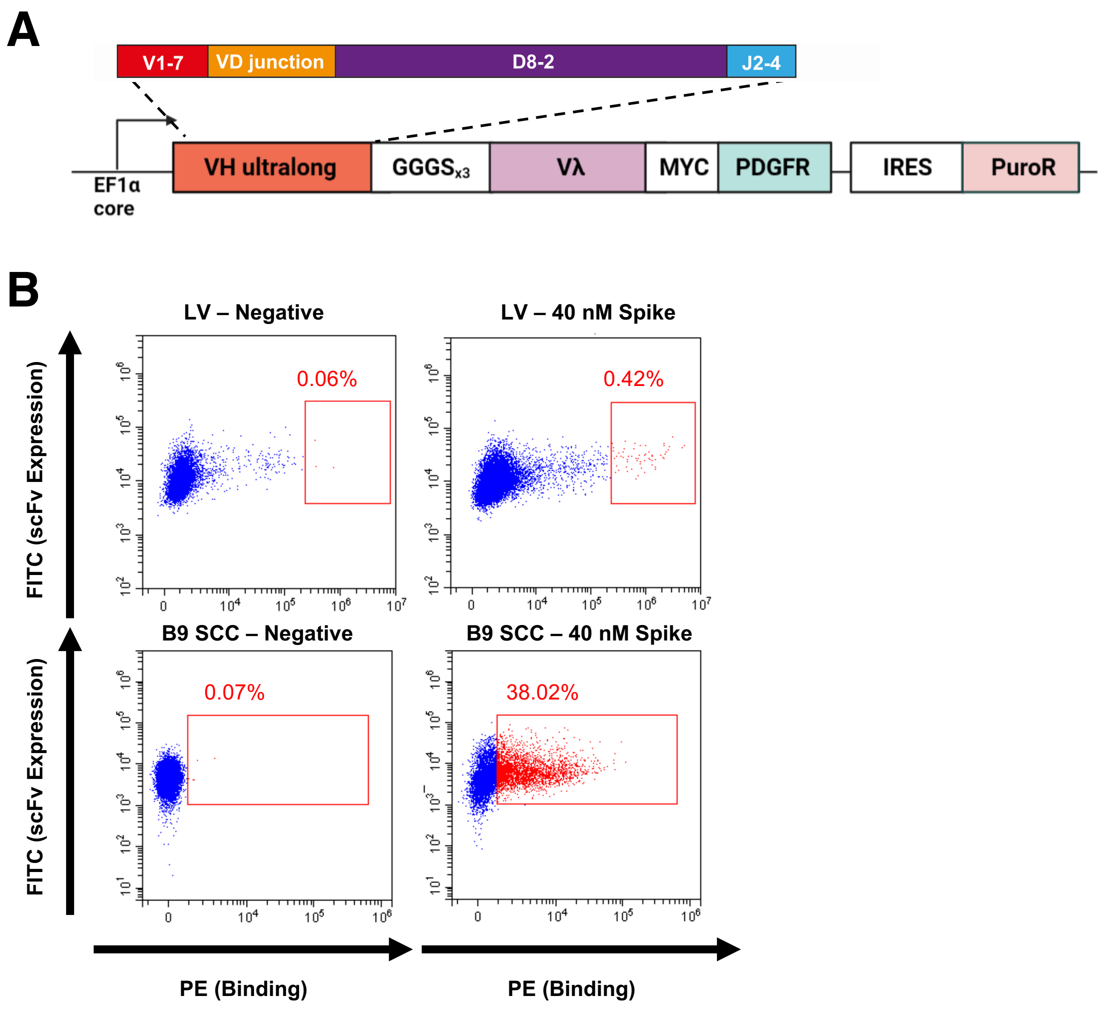
A
V1-7
VD junction
D8-2
J2-4
EF1α core
VH ultralong
GGGSx3
Vλ
MYC
PDGFR
IRES
PuroR
B
LV – Negative
LV – 40 nM Spike
0.06%
0.42%
B9 SCC – Negative
B9 SCC – 40 nM Spike
0.07%
38.02%
FITC (scFv Expression)
FITC (scFv Expression)
PE (Binding)
PE (Binding)

Fig. S2

Figure S2. Lentivirus transduction of scFv library. *A*, Cartoon showing the cloning of the enriched ultralong V_H library into the lentivirus vector used to generate stable cell lines. *B*, Upper: FACS plots showing stable polyclonal 293T cells transduced with enriched lentivirus scFv library incubated without (left) and with (right) 40 nM Spike. Lower: Single cell clone (SCC) B9-scFv incubated without (left) and with (right) 40 nM Spike. The red boxes show cells that express scFvs that bind Spike.

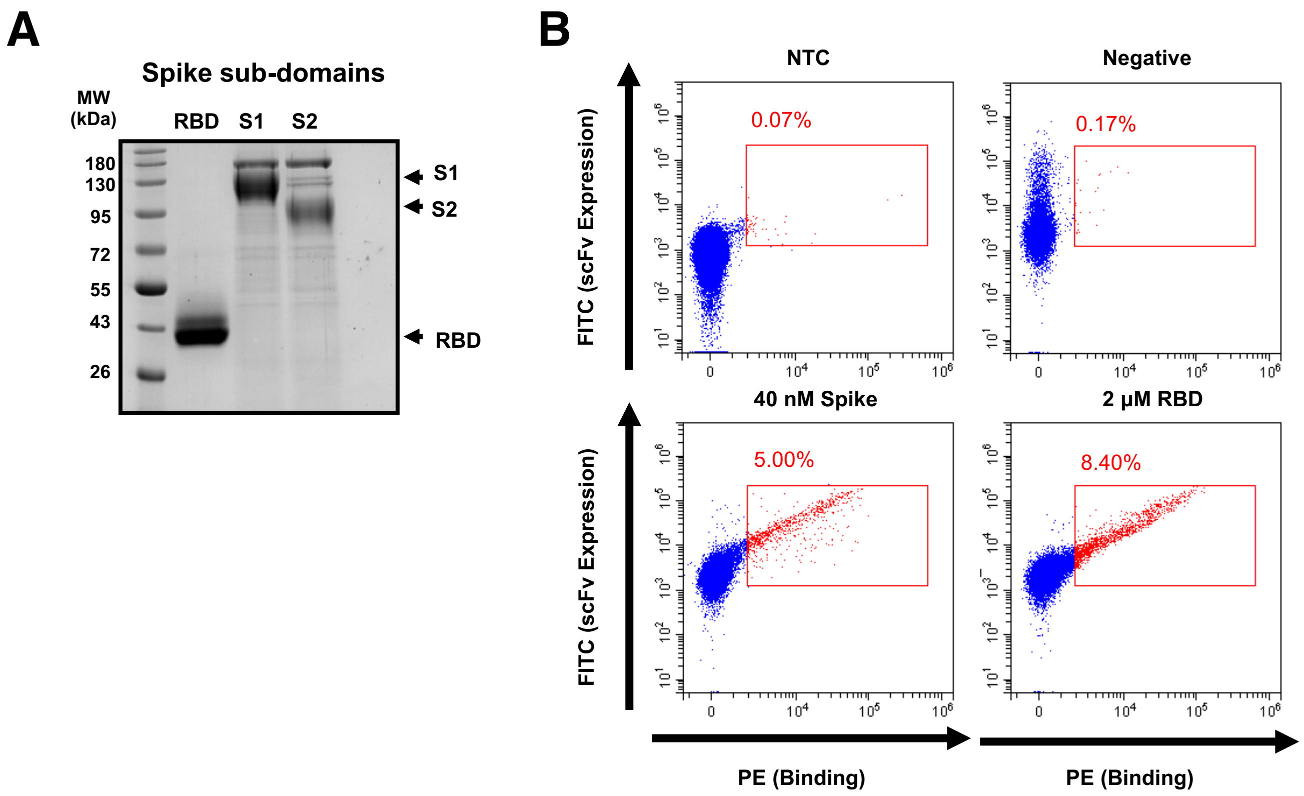
A
Spike sub-domains
MW
(kDa)
RBD
S1
S2
180
130
95
72
55
43
26
S1
S2
RBD
B
NTC
Negative
0.07%
0.17%
40 nM Spike
2 μM RBD
5.00%
8.40%
FITC (scFv Expression)
FITC (scFv Expression)
PE (Binding)
PE (Binding)

Fig. S3

Figure S3. Isolation of an ultralong scFv that binds to the wildtype SARS-CoV-2 Spike and receptor binding domain. *A*, SDS-PAGE of purified SARS-CoV-2 Spike sub-domains. *B*, FACS plots showing, Upper: non-transfected 293T cells (left) and 293T cells transfected with B9-scFV (right). Lower: 293T cells transfected with B9-scFv and incubated with 40 nM Spike (left) or 2 μM RBD (right).

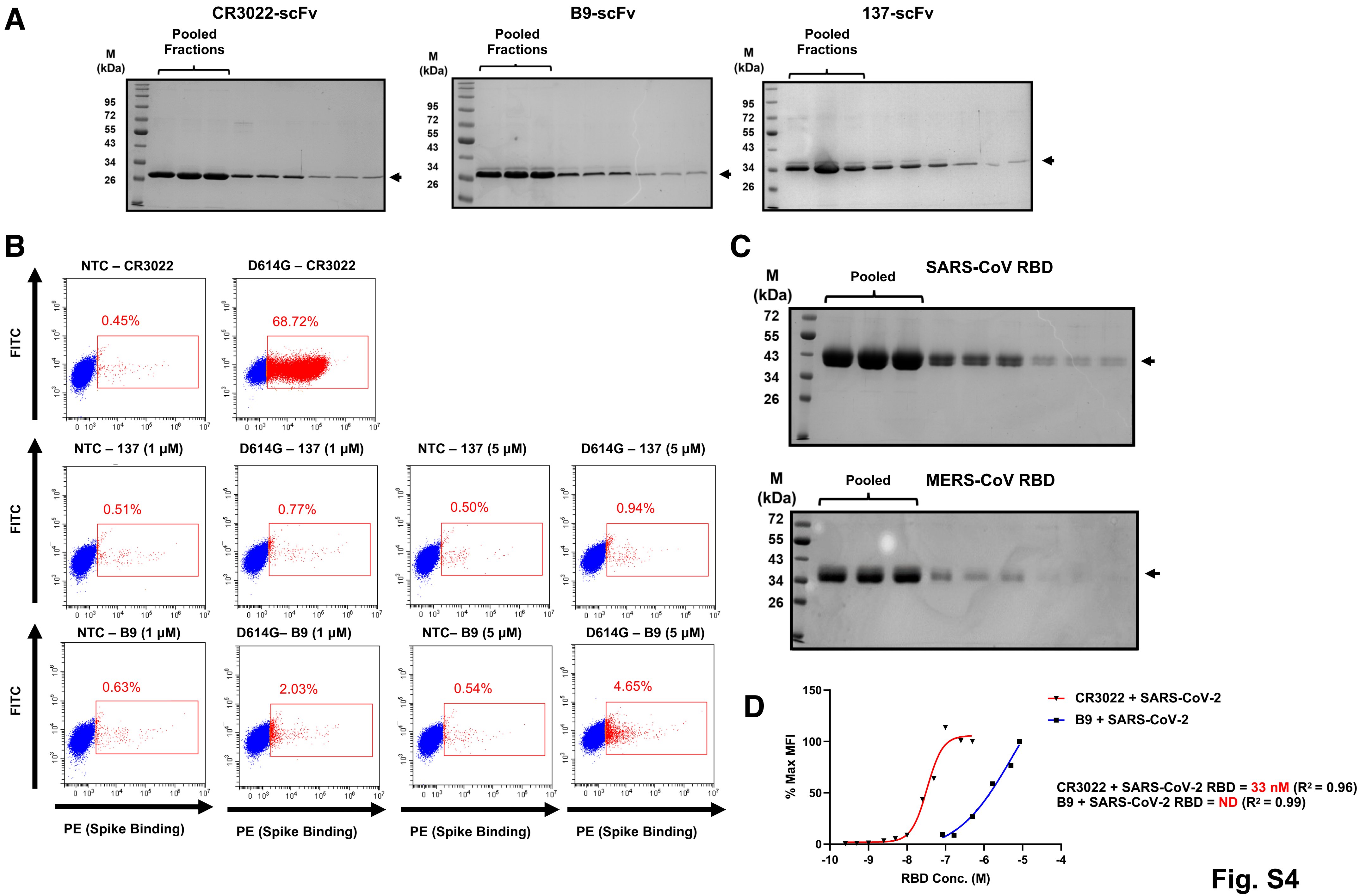
A
CR3022-scFv
B9-scFv
137-scFv
Pooled Fractions
M (kDa)
95
72
55
43
34
26
B
NTC – CR3022
D614G – CR3022
0.45%
68.72%
NTC – 137 (1 μM)
D614G – 137 (1 μM)
NTC – 137 (5 μM)
D614G – 137 (5 μM)
0.51%
0.77%
0.50%
0.94%
NTC – B9 (1 μM)
D614G– B9 (1 μM)
NTC– B9 (5 μM)
D614G – B9 (5 μM)
0.63%
2.03%
0.54%
4.65%
FITC
PE (Spike Binding)
C
SARS-CoV RBD
MERS-CoV RBD
Pooled
D
CR3022 + SARS-CoV-2
B9 + SARS-CoV-2
% Max MFI
RBD Conc. (M)
CR3022 + SARS-CoV-2 RBD = 33 nM (R² = 0.96)
B9 + SARS-CoV-2 RBD = ND (R² = 0.99)

Fig. S4

Figure S4. Purified B9-scFv binds to SARS-CoV-2 Spike expressed on the surface of 293T cells. *A*, SDS-PAGE gels of purified CR3022-, 137- and B9-scFvs. *B*, Upper: FACS plots showing non-transfected (NTC) and 293T cells expressing SARS-CoV-2 Spike (Wu-Hu-1+D614G) incubated with 200 nM CR3022-scFv to confirm cell surface Spike localization. Middle & lower: FACS plots showing non-transfected (NTC) and 293T cells expressing SARS-CoV-2 Spike protein (Wu-Hu-1+D614G) incubated with 1 and 5 μM 137-scFv (middle; negative control) or 1 and 5 μM B9-scFv (lower). Red box indicates scFv binding to 293T cells. *C*, SDS-PAGE of purified SARS-CoV (upper) and MERS-CoV (lower) RBD. *D*, 293T cells were transfected with B9-scFv or CR3022-scFv and incubated with increasing amounts of purified SARS-CoV-2 RBD. Estimated K_D and R^2 values are indicated.

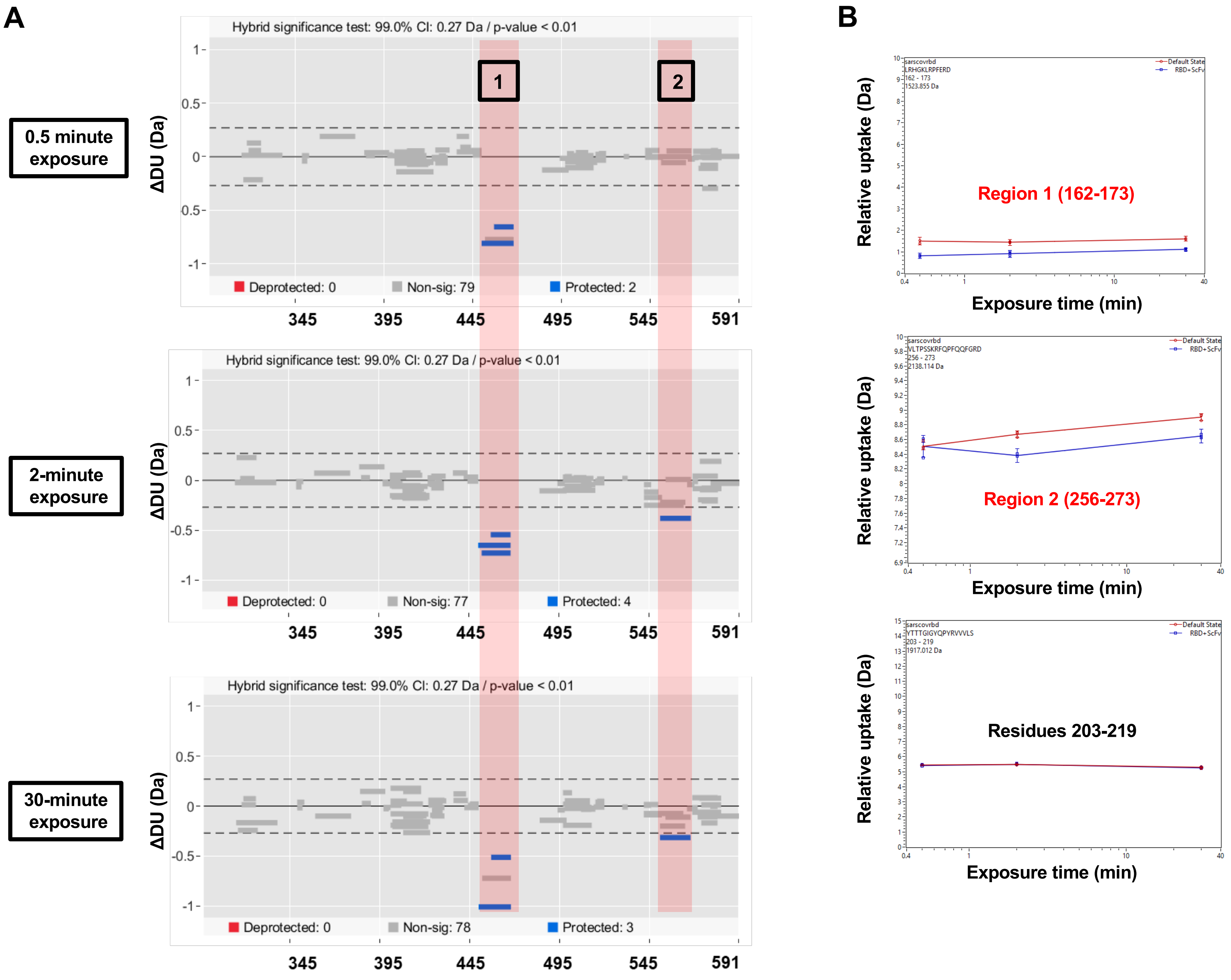

A
B
0.5 minute exposure
2-minute exposure
30-minute exposure
Hybrid significance test: 99.0% CI: 0.27 Da / p-value < 0.01
ΔDU (Da)
1
2
Deprotected: 0
Non-sig: 79
Protected: 2
Non-sig: 77
Protected: 4
Non-sig: 78
Protected: 3
345
395
445
495
545
591
Relative uptake (Da)
Exposure time (min)
Region 1 (162-173)
Region 2 (256-273)
Residues 203-219
Default State
RBD+ScFv
sarscovrbd
LRHGKLRPFERD
162 - 173
1523.855 Da
VLTPSSKRFQPFQQFGRD
256 - 273
2138.114 Da
YTTTGIGYQPYRVVVLS
203 - 219
1917.012 Da

Fig. S5

Figure S5. Hydrogen-deuterium exchange experiments for epitope localisation. *A,* Wood's plots showing the differences in deuterium uptake in SARS-CoV RBD at 0.5, 2 and 30 minutes of exposure to deuterium, comparing RBD alone to RBD in the presence of B9-scFv. Wood's plots were generated using Deuteros. Peptides coloured in blue are protected from exchange in the presence of B9-scFv and cluster to two regions (labelled 1 and 2 and highlighted in red). Peptides with no significant difference between conditions, determined using a 99% confidence interval (dotted line), are shown in grey. For each time point and condition, three replicate measurements were performed. *B,* Representative deuterium uptake plots of peptides derived from the RBD in region 1 (top), region 2 (middle) and a region where no protection from exchange was observed (bottom). Data are shown as mean ± standard deviation (n = 3).

A

Protein	Start	End	Peptide	State	Average uptake difference	Standard Deviation
SARS-CoV-RBD	449	467	YNYKYRYLRHGKLRPFERD	RBD + B9-scFv	-0.6526	0.0411
SARS-CoV-RBD	452	467	YKYRYLRHGKLRPFERD	RBD + B9-scFv	-0.7301	0.1911
SARS-CoV-RBD	456	467	LRHGKLRPFERD	RBD + B9-scFv	-0.5460	0.0810
SARS-CoV-RBD	551	565	VLTPSSKRFQPFQQFGRD	RBD + B9-scFv	-0.3821	0.0924

B

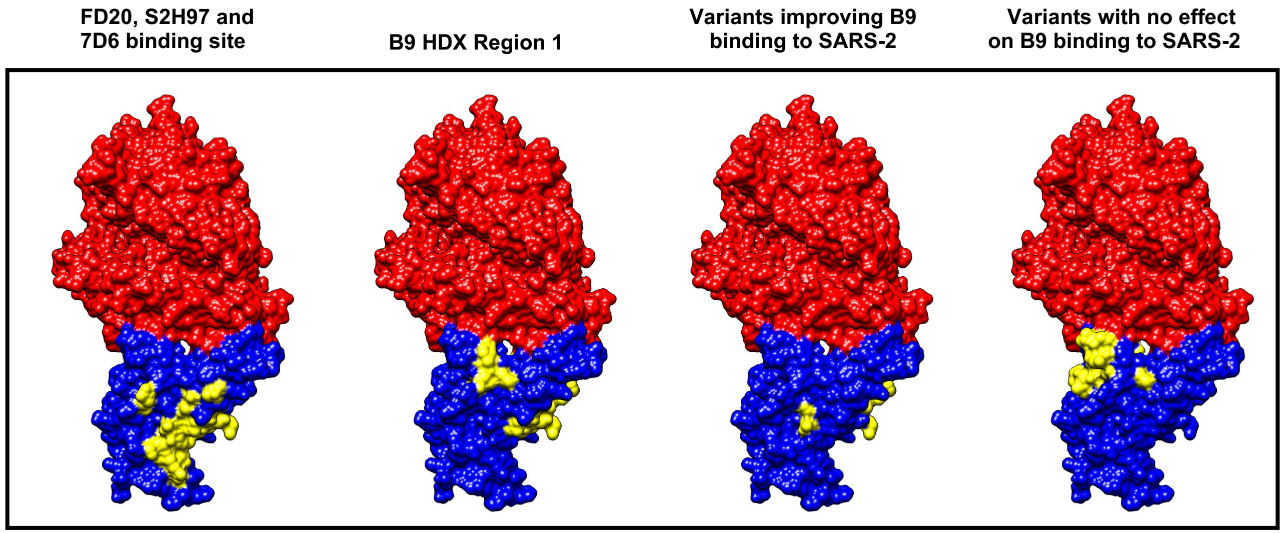

C

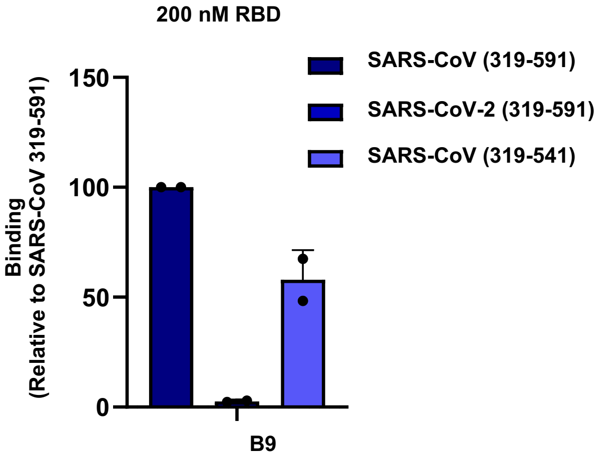

D

Mutations
A348P
N354E
P384A
Mut402-406 (I402V, R403K, E406D)
I434L
Mut438-447 (S438T, N439R, L441I, S443A, K444T, V445S, G446T)
L452K
Mut455-462 (L455Y, F456L, K458H, S459G, N460K, K462R)

Fig. S6

Figure S6. Localising the epitope of B9-scFv to a cryptic site by hydrogen-deuterium exchange and site-directed mutagenesis. *A*, Peptides of SARS-CoV RBD protected from deuterium uptake in the presence of B9-scFv following a 2-minute exposure to deuterated buffer. *B*, Surface maps of monomeric SARS-CoV-2 RBD (Blue) bound to hACE2 (red) generated using UCSF Chimera (PDB: 6M0J). Regions of interest are highlighted in yellow. There is significant overlap between the footprints of FD20, S2H97 and 7D6 with the proposed epitope of B9. *C*, 293T cells expressing B9-scFv on the cell surface were incubated with 200 nM of the recombinant RBD proteins shown. Binding was measured as the mean fluorescence intensity (MFI) and is presented relative to SARS-CoV (319-591). Data presented are mean ± SEM (n = 3). *D*, SARS-CoV-2 to SARS-CoV mutations tested in Fig. 5*C* and *D*.

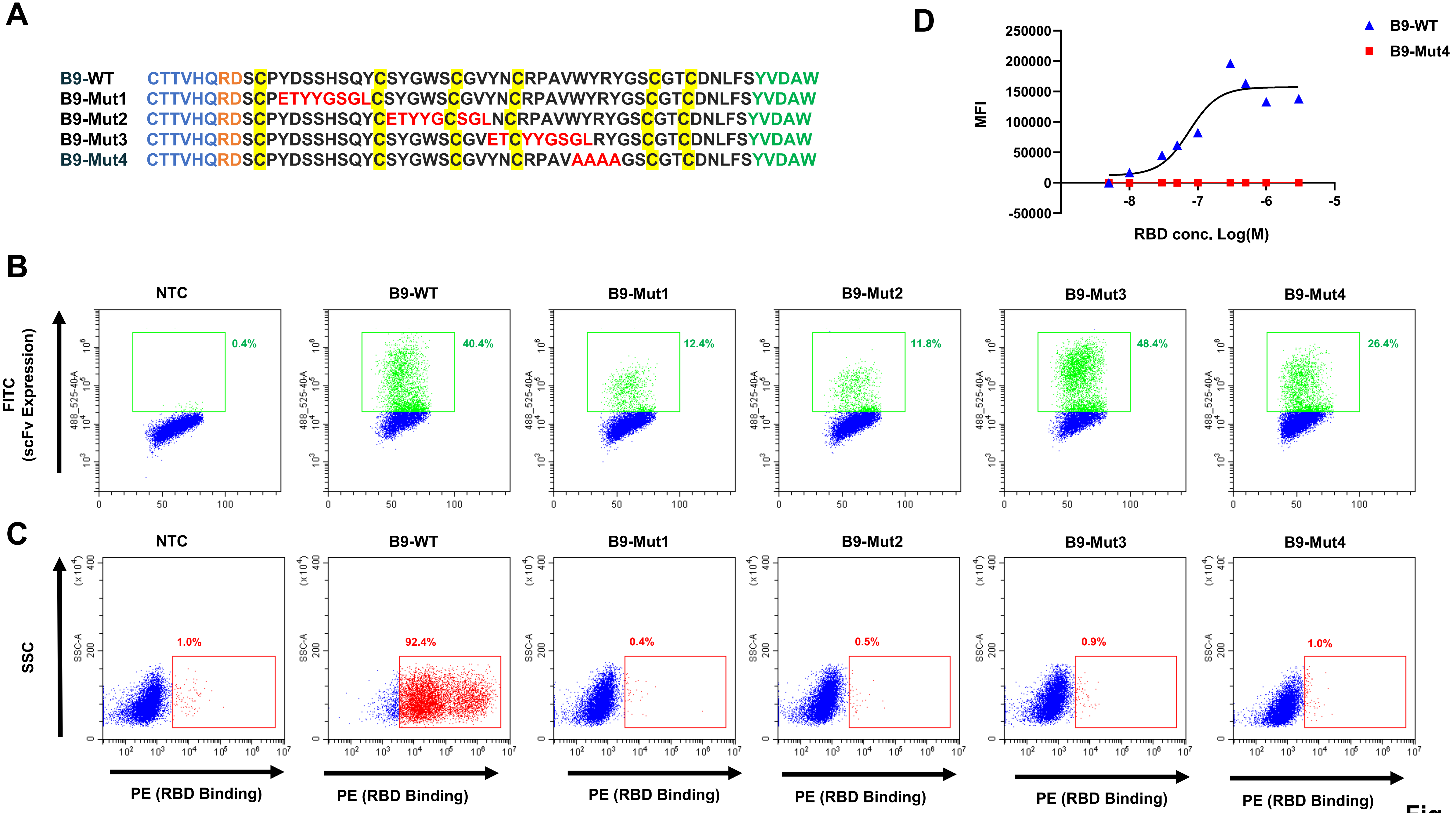
A
B9-WT CTTVHQRDSCPYDSSHSQYCSYGWSCGVYNCRPAVWYRYGSCGTCDNLFSYVDAW
B9-Mut1 CTTVHQRDSCPETYYGSGLCSYGWSCGVYNCRPAVWYRYGSCGTCDNLFSYVDAW
B9-Mut2 CTTVHQRDSCPYDSSHSQYCETYYGCSGLNCRPAVWYRYGSCGTCDNLFSYVDAW
B9-Mut3 CTTVHQRDSCPYDSSHSQYCSYGWSCGVETCYYGSGLRYGSCGTCDNLFSYVDAW
B9-Mut4 CTTVHQRDSCPYDSSHSQYCSYGWSCGVYNCRPAVAAAAGSCGTCDNLFSYVDAW
D
B9-WT
B9-Mut4
MFI
250000
200000
150000
100000
50000
0
-50000
-8
-7
-6
-5
RBD conc. Log(M)
B
FITC (scFv Expression)
NTC
B9-WT
B9-Mut1
B9-Mut2
B9-Mut3
B9-Mut4
0.4%
40.4%
12.4%
11.8%
48.4%
26.4%
488_525-40-A
0
50
100
C
SSC
NTC
B9-WT
B9-Mut1
B9-Mut2
B9-Mut3
B9-Mut4
1.0%
92.4%
0.4%
0.5%
0.9%
1.0%
SSC-A
(x 10⁴)
0
200
400
PE (RBD Binding)

Fig. S7

Figure S7. Mutagenesis of the B9 knob domain. *A*, B9 knob domain mutations tested for expression and RBD binding in (B). VH1-7, blue; VD junction, orange; DH8-2, black; JH2-4, green. Cysteines are highlighted in yellow, while amino acids mutated in B9-Mut1-4 are in red. *B,C* FACS plots showing the expression of B9-WT and B9-Mut1-4 scFvs (B) and their binding to 200 nM SARS-CoV RBD (C) as measured by FACS. SSC refers to side scatter. *D,* Binding of B9-WT and B9-Mut4 to a titration of SARS-CoV RBD (5 – 3000 nM). Binding is presented as mean fluorescence intensity (MFI). All samples were stained with α-Myc-FITC and α-His-PE (1:100) prior to analysis by flow cytometry. Expression (FITC) and binding (PE) are shown separately to improve clarity.

Table S1: Nucleotide sequences in the expression cassette.

Name	Sequence
pBovshow cassette	ATGGAGACAGACACACTCCTGCTATGGGTA<br>CTGCTGCTCTGGGTTCCAGGTTCCACTGGT<br>GACGCTGCTAGA-HeavyChain-<br>GGGATCTGGGGTGGTGGTTCTGGTGGTGG<br>TGGTTCTGGTGGTGGTGGTTCTTCGCGAGC<br>C-LightChain-<br>GAGGTCGACGAACAAAAACTCATCTCAGAA<br>GAGGATCTGAATGCTGTGGGCCAGGACAC<br>GCAGGAGGTCATCGTGGTGCCACACTCCTT<br>GCCCTTTAAGGTGGTGGTGATCTCAGCCAT<br>CCTGGCCCTGGTGGTGCTCACCATCATCTC<br>CCTTATCATCCTCATCATGCTTTGGCAGAAG<br>AAGCCACGTTAG
B9 Heavy chain variable domain	CAGGTGCAGCTGCGGGAGTCGGGCCCCAG<br>CCTGGTGAAGCCCTCACAGACCCTCTCGCT<br>CACCTGCACGGCCTCTGGATTCTCATTGAG<br>CGACAAGGCTGTAGGCTGGGTCCGCCAGG<br>CTCCAGGGAAGGCGCTGGAGTGGCTCGGT<br>GGTATAGACACTGGTGGAAGCACAGGCTAT<br>AACCCAGGCCTGAAATCCCGGCTCAGCATC<br>ACCAAGGACAACTCCAAGAGCCAAATCTCT<br>CTGTCAGTGAGCAGCGTGACAACTGAGGA<br>CTCGGCCACGTACTACTGTACTACTGTGCA<br>CCAACGAGATAGTTGTCCTTATGATAGTAG<br>CCATTCTCAGTACTGTTCTTACGGTTGGAGT<br>TGTGGTGTTTATAACTGTCGGCCTGCTGTTT<br>GGTATCGTTATGGTTCTTGTGGTACTTGTGA<br>TAATTTGTTCTCGTACGTCGATGCCTGGGG<br>CCAAGGACTCCTGGTCACCGTCTCCTCAGG<br>G
Vλ Light chain variable domain	CAGGCTGTGCTGAATCAGCCATCATCCGTG<br>TCCGGGTCCCTGGGCCAGAGGGTCTCCAT<br>CACCTGCTCTGGAAGCAGCAGCAATGTTGG<br>AAATGGATATGTGAGCTGGTACCAACTGAT<br>CCCAGGATCGGCCCCCAGAACCCTCATCTA<br>TGGTGACACCAGTCGAGCCTCGGGGGTCC<br>CCGACCGATTCTCCGGCTCCAGGTCTGGG<br>AACACAGCCACCCTGACCATCAGCTCGCTC<br>CAGGCTGAGGACGAGGCAGATTATTTCTGT<br>GCATCTGCTGAGGATAGTAGCAGTAATGCT<br>GTTTTCGGCAGCAGGACCACACTGACCGTC<br>CTGCTC

Table S2: HDX Data Summary Table. SD = standard deviation, CI = confidence interval.

**Data Set**	**SARS-CoV RBD**	**SARS-CoV RBD + B9-scFv**
**HDX reaction details**	50 mM potassium phosphate, pD 8, 0.3 M NaCl, 4 ºC	50 mM potassium phosphate, pD 8, 0.3 M NaCl, 4 ºC
**HDX time course (min)**	0.5, 2, 30 min	
**HDX control samples**	Maximally labeled controls were not performed.	
**Back-exchange**	~ 30 %	
**# of Peptides**	81	81
**Sequence coverage**	75.67%	75.67%
**Average peptide length / Redundancy**	11.04 / 3.94	11.04 / 3.94
**Replicates (biological or technical)**	3 (technical)	3 (technical)
**Repeatability**	0.0476 (average SD)	0.0465 (average SD)
**Significant differences in HDX (delta HDX > X D)**	Reference	Hybrid Significance test: 99% CI: 0.27Da/ p-value <-0.01
